# A stochastic model for identifying differential gene pair co-expression patterns in prostate cancer progression

**DOI:** 10.1186/1471-2164-10-340

**Published:** 2009-07-29

**Authors:** Wen Juan Mo, Xu Ping Fu, Xiao Tian Han, Guang Yuan Yang, Ji Gang Zhang, Feng Hua Guo, Yan Huang, Yu Min Mao, Yao Li, Yi Xie

**Affiliations:** 1 State Key Laboratory of Genetic Engineering, Institute of Genetics, School of Life Science, Fudan University, Shanghai 200433, PR China; 2 Shanghai Biostar Gene company, Shanghai 200437, PR China

## Abstract

**Background:**

The identification of gene differential co-expression patterns between cancer stages is a newly developing method to reveal the underlying molecular mechanisms of carcinogenesis. Most researches of this subject lack an algorithm useful for performing a statistical significance assessment involving cancer progression. Lacking this specific algorithm is apparently absent in identifying precise gene pairs correlating to cancer progression.

**Results:**

In this investigation we studied gene pair co-expression change by using a stochastic process model for approximating the underlying dynamic procedure of the co-expression change during cancer progression. Also, we presented a novel analytical method named 'Stochastic process model for Identifying differentially co-expressed Gene pair' (SIG method). This method has been applied to two well known prostate cancer data sets: hormone sensitive versus hormone resistant, and healthy versus cancerous. From these data sets, 428,582 gene pairs and 303,992 gene pairs were identified respectively. Afterwards, we used two different current statistical methods to the same data sets, which were developed to identify gene pair differential co-expression and did not consider cancer progression in algorithm. We then compared these results from three different perspectives: progression analysis, gene pair identification effectiveness analysis, and pathway enrichment analysis. Statistical methods were used to quantify the quality and performance of these different perspectives. They included: Re-identification Scale (RS) and Progression Score (PS) in progression analysis, True Positive Rate (TPR) in gene pair analysis, and Pathway Enrichment Score (PES) in pathway analysis. Our results show small values of RS and large values of PS, TPR, and PES; thus, suggesting that gene pairs identified by the SIG method are highly correlated with cancer progression, and highly enriched in disease-specific pathways. From this research, several gene interaction networks inferred could provide clues for the mechanism of prostate cancer progression.

**Conclusion:**

The SIG method reliably identifies cancer progression correlated gene pairs, and performs well both in gene pair ontology analysis and in pathway enrichment analysis. This method provides an effective means of understanding the molecular mechanism of carcinogenesis by appropriately tracking down the process of cancer progression.

## Background

Microarray technology enables us to examine the expressions of thousands of genes on a genomic scale. It has great potential to reveal the molecular mechanism of many diseases including cancer progression. However, because microarray technology usually uses differentially expressed genes as its fundamental base, it generates an enormous amount of information. Analysing this large amount of information for researchers is strenuous and often leaves to miss accurate interpretation of it.

Recent studies based on the pattern of gene co-expression shed light on the deficient methods to analyse the expression of differential genes. Evidence has shown that genes with similar transcriptional expression profiles are likely to be regulated through the same mechanisms [1]. This pattern change of co-expression at the transcriptional level may directly indicate the change of regulatory networks during different stages of cancer progression. There also exists a relationship between gene pair co-expression and the interaction of their encoded proteins [2-5]. This protein interaction variation is able to be monitored on a genomic scale via the change of gene pair co-expression. Furthermore, analysis of genome-wide co-expression may provide information on those weakly expressed differential genes. Nevertheless they are co-expressed with detectable differentially expressed genes [6].

Several approaches [7-10] have been made to explore differential gene pair co-expression patterns at two biological stages. Lai YL *et al*. [7] extended the traditional F-statistic to Expected Conditional F-statistic (ECF-statistic), to detect these gene pairs at different cellular states. Choi JK *et al*. [8] combined the effect size as a standardized index for meta-analysis, to measure the covariate effect of gene pairs in a number of different cancers, then constructed cancerous and healthy co-expression networks; with the view of gene network, differentially co-expressed gene pairs could be identified. Li KC [10] devised a conception of liquid association (LA) to study genome-wide co-expression dynamics. His method shows that the co-expression alteration of two genes depends on the expression level of a third gene. Yoon SH *et al*. [9] defined a correlation ratio to present gene pair co-expression change and assessed statistical significance for gene co-expression change based on a distribution of correlation ratio.

All of the above methods are very insightful; however, they lack to provide a view that the change of gene pair co-expression pattern results from disease progression. Identifying gene co-expression pattern change usually involves a statistical significance assessment. If a cancer progression is not considered during the assessment of significance, the identified gene pairs would have less correlation with cancer progression. On the other hand, some gene pairs representing disease progression might be missed. Therefore, more realistic model concerning cancer progression in a significance assessment is needed.

A stochastic process model is an ideal tool to address progression-related issues. For a series of random events, the stochastic process has the capability to quantify the inherent dynamic rules in terms of probability; it is certainly being useful when uncertainty is involved in the progression.

Cancer progression is an evolutionary process which is constituted by a series of random genomic mutation events and governed by selective pressure [11]. Under such pressure the mutations acquired result in a relatively small number of clones of cancer cells [12]. Such mutated cells genetically develop into being immune evasive, resistant to chemotherapy, and very adaptive to androgen ablation for a prostate cancer. These nature selected mechanisms for survival result from random events presented in the final consecutive genetic alterations. During recent decades, a number of stochastic models [12-14] with varying degrees of mathematical complexity have been presented to describe some processes of carcinogenesis, such as cell growth, chemotherapy resistance, and angiogenesis.

To analyse co-expression performance during cancer progression, understanding that at each mutation step the co-expression of a gene pair may change randomly is critical. Therefore, in our study we mainly seek to develop a stochastic system to study the gene pair co-expression change. During the process, most co-expressions do not change remarkably. Yet, some co-expressions of genes have significant changes which are determined or elected by nature selection during cancer cell evolution. Our method uses the theory of evolution as foundation for significance assessment. The stochastic process is used to approximate the co-expression change as a null distribution based to identify significant co-expression change. This identification process highly correlates with cancer progression procedure, and the identified gene pairs could reflect nature selected cancer cell's strategy for survival. In particular, there is a simple stochastic process case – the random walk, which has been widely used in carcinogenesis study, especially when genomic variations are involved [15-17]. Since gene pair co-expression changes take place on a genomic scale, in this study we employ the random walk model to approximate co-expression change during cancer progression.

Prostate cancer is a leading cause of death in the United States and Western Europe [18]. Dietary factors, lifestyle-related factors, and hormones, particularly androgens, have long been implicated in the pathogenesis of prostate cancer (review [19]). Therapy for the advanced form of prostate tumour generally involves either surgical gonadectomy to remove the major source of androgens or drug treatments that suppress androgen production and transportation. These treatments are somewhat effective because they can initiate apoptosis in prostate cancer cells. When the tumour is able to be effectively treated it is termed as being hormone sensitive (HR). Unfortunately, some fractions of the prostate cancer cells finally survive the therapy, and become highly aggressive and metastatic. This treatment-ineffective stage is usually regarded as hormone refractory (HR). The molecular mechanisms of prostate cancer carcinogenesis and progression are still hard to understand in part due to the few studies focusing on detecting gene co-regulation changes in the context of cancer progression.

In this study, we present a novel analytical method named 'Stochastic process model for Identifying differentially co-expressed Gene pair' (SIG method) to investigate gene pair co-expression change. The SIG method aims to identify gene pairs with significant differential co-expression patterns, and combines a stochastic model with gene pair co-expression change. For each gene, we derive its analytical distribution of co-expression change with other genes; which is very useful when wanting to determine the significance assessment of gene pair co-expression change. Based on analytical distribution results and multiple hypothesis testing, we assess the significance of co-expression change. Furthermore, we are then able to identify gene pairs significantly different co-expressed. We apply this method to two microarray data sets, both of which measure gene expressions in prostate cancers with progression relationships. One data set consists of hormone sensitive tumours and hormone refractory tumours [20], the other data set consists of healthy and cancerous prostate tissues [21]. When the SIG method is applied a large number of gene pairs co-expressed differentially are then detected, and some regulatory networks are reconstructed according to the identified pairs. In turn, many mechanisms of prostate cancer progression are revealed. This method is compared with two other similar methods: the Expected Conditional F-statistic method (ECF method) presented by Lai YL *et al*. [7] and the Detection of Altered Gene Associations method (DAGA method) proposed by Yoon SH *et al*. [9]. To evaluate the methods systematically, we compare the results of three methods from three different perspectives: cancer progression analysis, gene pair analysis, and pathway analysis.

## Results

Being stochastic model based, the SIG method is able to calculate and predict the process of co-expression change. The effect of cancer progression is involved in gene pair identification, and gene pairs with significant co-expression change could be detected with high relevance to cancer progression. Herein, the three approaches of the SIG method, the ECF method [7] and the DAGA method [9] were applied to two data sets [20,21], and their performance are assessed comparably from three perspectives: progression, gene pair and pathway. The same threshold for significance assessment was set for the three methods, and results of performance comparison were listed in table 1.

**Table 1:** Performance comparison for three methods

**Data type**	**Statistics**	**Methods**		
		
		SIG method	ECF method	DAGA method
***HS ***vs. ***HR***	Identified pairs	428,582	2,101,425	5,212,394
	RS	1.49%	100%	91.07%
	P-value of RS	0.011	1	0.93
	PS_Avg	1.74	0	0.02
	P-value of PS_Avg	0.032	1	0.97
	TFR	1.39%	0.49%	0.26%
	PES_Avg	3.56	2.56	2.02

***cancer ***vs. ***healthy***	Identified pairs	303,992	14,228,478	32,569,223
	RS	1.40%	100%	96.21%
	P-value of RS	0.013	1	0.87
	PS_Avg	1.65	0	0.01
	P-value of PS_Avg	0.036	1	0.99
	TFR	0.92%	0.39%	0.25%
	PES_Avg	3.32	2.36	1.86

RS: Re-identification Scale, overlap between the pairs identified before and after changing disease progression relation; PS: Progression Score, ratio of the nominal P-value of a pair before changing disease progression relation to that of a pair after changing disease progression relation; TPR: True Positive Rate, the proportion of functionally relevant pairs in all characterized pairs; PES: Pathway Enrichment Score, the degree to which differentially co-expressed gene pairs are overrepresented at a pathway. The 'Avg' refers to the average value for an item.

### Datasets used

Two public microarray data sets were used to assess the performance of three methods. One data set [20] includes 10 HR primary prostate tumour biopsies and 10 primary untreated HS tumours. After dissecting the samples by Laser Capture Microdissection, the RNA was extracted and amplified. The gene expressions were assessed using Affymetrix Human Genome U133A GeneChip, which contains probes representing 22,344 genes and expressed sequence tags (ESTs). We normalized the data by rescaling all arrays in order to equalize the median intensities of them. For each chip, the intensities of replicated genes were averaged. Genes with more than 60% missing values were excluded. The missing values of remaining genes were imputed via KNN method which uses nearest neighbour averaging. After filtering, there were 4,848 genes for analyses.

The other data set [21] without missing value contains high-quality expression profiles which were derived from 52 prostate tumours and 50 healthy prostate specimens, using oligonucleotide microarrays containing probes for approximately 12,600 genes and ESTs. The methods for normalization and processing replicated genes were the same as those for the above data set and 8,921 genes remained after data pre-processing.

### Analysis from progression perspective

Generally, cancer progression is irreversible. For instance, prostate cancer progresses from HS stage into HR stage; however, HR prostate cancer hardly converts back into HS prostate cancer. Therefore, taking the progression of cancer into account, one will tend to identify the gene pair highly correlated with progression. We call such gene pair as 'progressive'. In contrast, given a reverse progression the possibility of identifying this gene pair would be eliminated.

First step was to calculate the gene pair differential co-expression based on the original order of progression, the identified gene pairs were then called 'pre-changed' pairs. Next, we reversed this data invertedly to the order of progression. These identified gene pairs were then called 'post-changed' pairs. This identification procedure was also performed when using the other two methods. We defined two metrics to investigate the reliability of a method for identifying progressive gene pairs: the Re-identification Scale (RS), and the Progression Score (PS).

To examine the overlap between pre-change pairs and post-change pairs, a metric named Re-identification Scale (RS) was defined:

(1)

where *N*_overlap _represents the number of overlapped gene pairs, and *N*_pre _represents the total number of pre-change pairs. The smaller the RS value, the better the method for detecting gene pairs relevant to cancer progression. The statistical significance of a RS value was calculated using one-sided random permutation test. The RS value for the real data was calculated and compared with 1,000 RSs generated by randomly assigning the sample labels to the expression values of the genes. For each permutated data set, pre-change pairs and post-change pairs can be identified under the above procedure using the SIG method. The nominal P-value then is the fraction of RS values of random data that were smaller than or equal to the RS value of real data.

As illustrated in table 1, the RSs obtained using the SIG method are less than 2% of the two data sets and are highly significant, suggesting that the gene pairs identified by our method are 'progressive'. While the other two methods are not designed to detect progressive gene pairs, which leads to the RS values all close to 1 with little significance.

In order to make the progression grade for each gene pair, we defined a metric named Progression Score (PS):

(2)

where *P*_pre _and *P*_post _correspond to the nominal P-values of pre- and post- progression change, respectively. We averaged the PSs of all gene pairs and used the PS average as a metric to compare the three methods. The significance of a PS average was calculated similarly to preceding RS significance assessment using one-sided random permutation test. The significance P-value is the fraction of PS average of random data that were greater than or equal to the PS average of real data.

Using the ECF method and the DAGA method, the *P*_pre _and *P*_post _of each gene pair are almost equal as predicted. Accordingly, the PSs for all gene pairs identified by the two methods are close to 0 and have little significance (Table 1). In contrast, the SIG method has high PS average values which are far from 0 with P-values of 0.032 and 0.036 for the two data sets.

**Figure 1:**
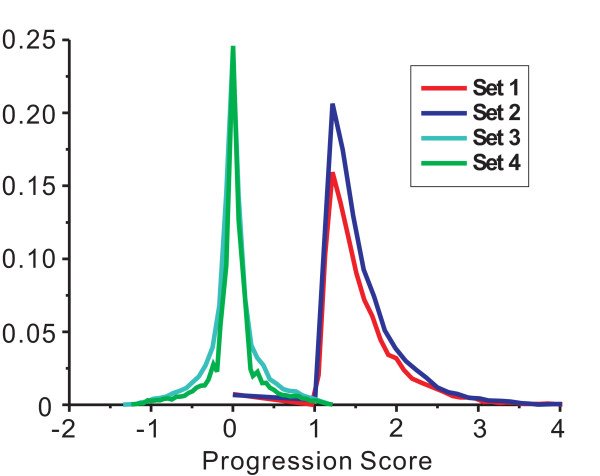
**Progression score (PS) distribution of all gene pairs**. Set1 refers to the gene pairs both identified by the SIG method and the ECF method; Set 2 contains gene pairs exclusively identified by the SIG method; Set 3 contains gene pairs cannot be identified by the SIG method; Set 4 contains gene pairs exclusively identified by the ECF method. Gene pairs in Set 1 and Set 2 show much higher PS values compared with the gene pairs in other two Sets.

To investigate whether gene pairs identified by the SIG method are more relevant to cancer progression than those identified by other methods, we calculated the distribution of PSs of identified gene pairs (Figure 1). By applying the SIG method and the ECF method to the HS cells versus HR cells data set, we calculated PS distribution for four sets of the results. Set 1 contains gene pairs identified both by the SIG and the ECF methods. Set 2 contains gene pairs exclusively identified by the SIG method. Set 3 contains gene pairs not identified by the SIG method. Set 4 contains gene pairs exclusively identified by the ECF method. The SIG algorithm was then applied to the four gene pair sets to calculate Progression Scores. All of the four calculated PS distributions are illustrated in Figure 1. A great majority of gene pairs in Set 1 and Set 2 show much higher PS values when comparing them with the values of the gene pairs in Set 3 and Set 4, in which PSs are around 0. This suggests that the SIG method identifies progressive gene pairs. The PS distribution of Set 4 is almost the same as that of Set 3, indicating that the gene pairs solely identified by the ECF method show less relevance to the cancer progression.

Additionally, some gene pairs have only been identified by the SIG method, such as *CEBPD *and *CTNNB1*, *FAS *and *FADD*, *BCL2L2 *and *BID*. These genes are involved in the progression from HS stage to HR stage; with high PS significance, their nominal P-values of PS are less than 0.05. Understanding how a pair of genes is involved in prostate cancer progression is possible, because the interaction of two proteins links with the co-expression pattern of the genes encoding them [2-5]. The correlation coefficients and PS values of the gene pairs in this study are listed in additional file 1.

Gene pair of *CEBPD *and *CTNNB1 *shows strong negative correlation at HR stage and low at HS stage. *CEBPD *encodes a transcription factor important for adipogenesis in promoting preadipocyte's differentiation. *CTNNB1 *encodes β-catenin which plays a key role in Wnt signalling, and maintains preadipocytes at an undifferentiated state through inhibition of *CEBP*s [22]. Compared with HS stage, cancer cells at HR stage are poorly differentiated and thus *CEBPD *is more inclined to the inhibition of *CTNNB1*. Therefore, this pair has negative correlation at HR stage, and reveals a clue for cell progression to an undifferentiated status at HR stage.

*FAS *and *FADD *are apoptosis-related genes. *FAS *encodes a death receptor, *FADD *encodes a death domain-containing adaptor, and activated *FAS *can recruit *FADD *by homophilic interaction [23]. The result shows that correlation of *FAS *and *FADD *is positively high at HR stage and low at HS stage. The HS samples are untreated, still fed with androgens; the HR cancer cells are androgen ablation treated and deprived of 'nutrition'. Therefore, more apoptosis has been initiated for HR cells, which results in *FAS *and *FADD *only highly correlating at the HR stage and initiating an apoptosis pathway. These early actions take place as a step for prostate cancer progressing from HS stage to HR stage.

Gene pair of *BCL2L2 *and *BID *shows highly negative correlation at the HR stage and low correlation at the HS stage. *BCL2L2 *contributes to reduced cell apoptosis, and *BID *encodes a death agonist with physical binding to *BCL2L2 *[24]. In HR stage, apoptosis has been initiated by *FAS *– *FADD *interaction, but most cells still survive. This implies apoptosis may be blocked in the downstream of this pathway. Our result suggests that *BCL2L2 *may bind *BID *to mask its pro-apoptotic activity and to block the initiated apoptosis at the HR stage. Therefore, this information reveals a critical step in the progression of prostate cancer, how some cells are not driven by apoptosis.

### Analysis from gene pair perspective

As many gene pairs may be identified, we ask whether these pairs are of biological significance. To address this issue, a True Positive Rate (TPR) was defined to assess the biological reliability of the identified pairs. Two genes can either be functionally relevant with True Positive (TP) or irrelevant with False Positive (FP). To quantify the function relevance of identified gene pairs, we used curated pathway annotations from the KEGG, GenMAPP databases, and GO annotations. When two genes share the same pathway annotation in any of the databases, the pair is labelled as TP; when two genes are annotated with two non-overlapping pathways this pair is labelled as FP. In some cases one gene of a pair is not annotated, such gene pairs are defined as non-discriminatory (ND) since current knowledge is insufficient to conclude whether they are functionally relevant or not. We then defined True Positive Rate (TPR) as:

(3)

As shown in Table 1, the number of identified gene pairs by our method is the lowest of the three methods. The number of gene pairs identified using the SIG method constitutes only about 20% of the pairs using the ECF method, and 10% of the pairs using the DAGA method. The TPR values of the SIG method are much higher than those of other two methods, demonstrating that our method is more effective in selecting biologically relevant gene pairs.

**Figure 2:**
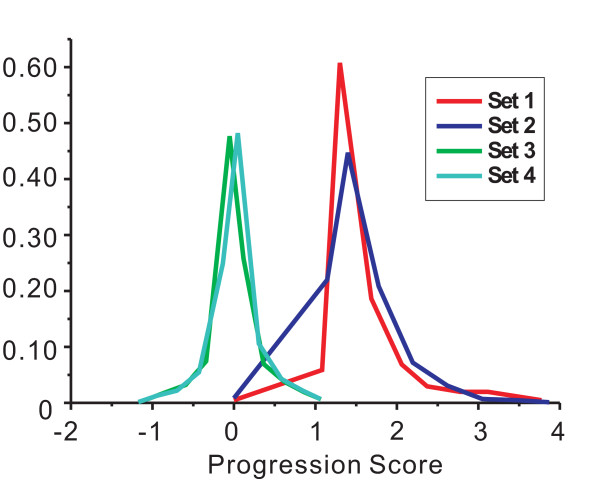
**Progression score (PS) distribution for True Positive (TP) gene pairs**. Set 1 refers to the TP gene pairs both identified by the SIG method and the ECF method; Set 2 contains TP gene pairs exclusively identified by the SIG method; Set 3 contains TP gene pairs cannot be identified by the SIG method; Set 4 contains TP gene pairs exclusively identified by the ECF method. TP gene pairs in Set 1 and Set 2 show the higher PS values compared with the other two sets.

Additionally, the TP gene pairs identified by different methods were investigated with Progression Score to determine whether they were involved in prostate cancer progression. Similar to the procedure to determine total gene pairs' PS distribution, we took HS cell data versus HR cell data as the specimen sample, and used the ECF results for comparison. We calculated Progression Score (PS) distributions for four TP gene pair sets. As shown in Figure 2, TP pairs in Set 1 and Set 2 have much higher PS values compared with TP pairs in Set 3 and Set 4. The PS distributions of TP pairs in Set 3 and Set 4 are both low and very close to each other. The PS distribution difference between Set 2 and Set 4 implies that the 'true positive' gene pairs identified exclusively by the ECF method demonstrate little relevance to cancer progression; furthermore, the TP gene pairs identified by the SIG method have stronger correlation with progression, suggesting that they are more likely involved in cancer development.

### Analysis from pathway perspective

Pathway information is vital for successful function analysis of cancer progression. A pathway is a set of gene-gene co-regulations which operate in concert to carry out a biological process and typically present abnormalities in molecular disease mechanisms [25]. Therefore, for a given pathway, we can investigate how it is enriched to affect cancer progression with identified differentially co-expressed gene pairs. In this context, we perform pathway enrichment analysis to determine cancer progression related pathways.

To investigate the ability of a method to identify progressive functional relevant pathways based on enrichment analysis, we formulated a Pathway Enrichment Score (PES) that reflects the degree to which differentially co-expressed gene pairs are overrepresented in a pathway. The calculation of the PES was similar to the Gene Set Enrichment Analysis [26]. We ranked all identified gene pairs according to their metric of ordered co-expression change. For any given pathway, a score was calculated by reading in a downward direction in the pair list. When we encountered a pair of genes located both in this pathway, the score increases; otherwise, it decreases. The PES is calculated as the maximum value deviated from zero. The higher a PES value, the more identified gene pairs enriched in a pathway.

Totally 144 previously studied pathways were downloaded from the KEGG and the GenMAPP databases and chosen for the PES calculation. These PES results were calculated using the three methods (the SIG method, the ECF method, and the DAGA method), and are listed in additional file 2.

It is known that a large number of pathways are involved in prostate cancer progression. Thus, we measured the functional involvement of all pathways to compare global effects of the three methods. For the data set which shows progression from healthy to cancerous, the average PES values of all pathways combined were 3.32, 2.36, and 1.86 calculated by the SIG method, the ECF method, and the DAGA method, respectively (Table 1). The P-values of the t-test were 2.5E-03 and 1.18E-09, for the PES value difference between the SIG method versus the ECF method, and for the PES value difference between the SIG method versus the DAGA method, respectively. For the data set with progression from HS classification to HR classification, the PES averages were 3.56, 2.56 and 2.02 (Table 1), respectively. The P-values of the t-test were 2.5E-03 and 1.74E-09, for the PES value difference between the SIG method versus the ECF method, and for the PES value difference between the SIG method versus the DAGA method, respectively. The highest PES average value obtained by the SIG method indicates that among the three methods, our method shows the highest capability to identify functional pathways relevant to progression.

**Figure 3:**
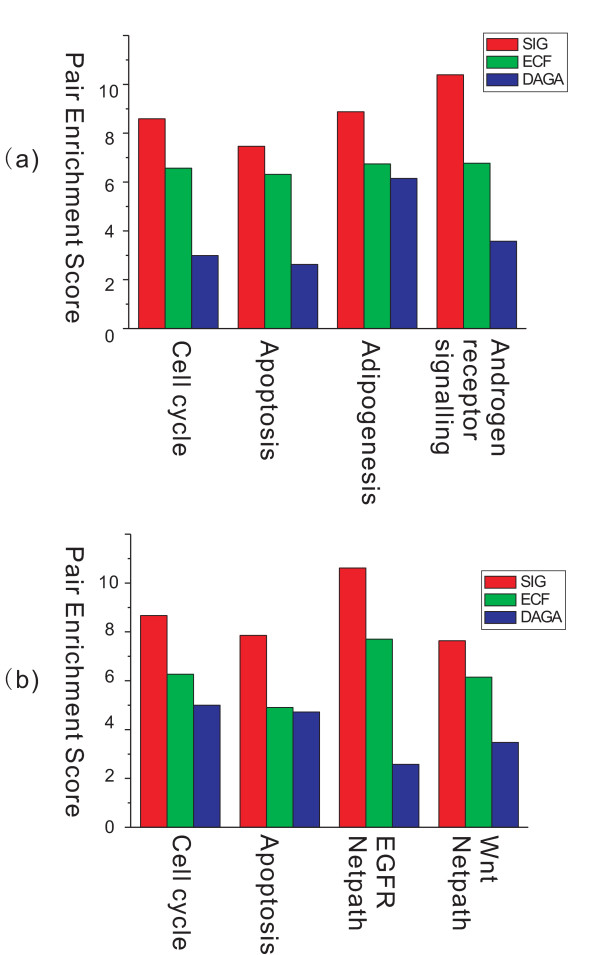
**Pathway enrichment score (PES) comparison**. Pathways known to be involved in the progression of prostate cancer are employed. (a) The four pathways involved in prostate cancer progression from HS stage to HR stage. (b) The four pathways involved in prostate cancer progression from healthy stage to cancerous stage.

Best CJ *et al*. [20] reported that several pathways including cell cycle, apoptosis, adipogenesis, immunoreaction coordination and androgen receptor signalling, have significant effects in distinguishing the HR state from the HS state, and playing important roles in prostate cancer progression. In addition, Singh D *et al*. [21] suggested that the pathways of cell cycle, apoptosis, angiogenesis, epidermal growth factor receptor (EGFR) netpath and Wnt netpath, are involved in deciding the progression process from healthy to cancerous. For each individual pathway mentioned above, we compared the PES values obtained from the three methods. As illustrated in Figure 3, the SIG method showed the highest PES values for all the mentioned pathways in the two data sets. Since these pathways have been reported as progression related, it is suggested that the SIG method is suitable for detecting pathways associated with cancer progression in comparison with other two methods.

## Discussion

We compared the SIG method with the ECF method and the DAGA method by applying them to two different data sets [20,21]. After the investigation of performance, our method shows stronger reliability in identifying progressive gene pairs and progression relevant pathways. In addition to its convincing performance, the SIG method has several remarkable features in its algorithm (Table 2). Moreover, based on the gene pairs identified by the SIG method, we can not only deeply interpret some previously unknown interactions between a pair of genes, but also infer several gene interaction networks involved in the mechanisms of prostate cancer progression.

**Table 2:** Algorithm comparison for three methods

**Features**	**Methods**		
	
	SIG method	ECF method	DAGA method
Measure of co-expression change	z-CC ratio	ECF-statistic	z-CC ratio
Analytical distribution	+	-	+
Progression consideration	+	-	-
Multiple test analysis	+	-	-
Gene specificity analysis	+	-	-
Gene pair duple confirmation	+	+	-

The symbols '+' and '-' represent existence and absence of the corresponding categories. Because the distribution for each gene is different, gene pair duple confirmation refers to a gene pair (A, B) could be identified only when gene A is chosen, the pair shows significant co-expression change; meanwhile, when gene B is chosen, the pair is also determined with significant differential co-expression between two progressive stages.

### Algorithm features

First of all, in our method, the co-expression change between different cancer stages is measured by the ratio of co-expression. Then the analytical distribution of correlation ratio was derived using stochastic process model to work as the foundation of significance assessment. Therefore, the nominal P-value of the gene pair being concerned could be calculated. In the ECF method, an ECF-statistic was used as a metric for gene pair co-expression change analysis. To estimate its significance level, an approximate distribution for ECF-statistic was obtained with a large number of simulation data to make up the null hypothesis. It gives rise to two questions: even if the number of simulations is large, the distribution is still skewed from biologically structured distribution; genes play a variety of roles *in vivo *in terms of the range of genes they influence directly and indirectly. A transcription factor in upstream of a pathway may regulate many target genes. In contrast, a gene in downstream of a pathway may only interact with a few other genes. Thus, genes should be considered specifically, instead of being uniformly treated. The DAGA method also derived the theoretical distribution of correlation ratio; while it lacks the factors of cancer progression and gene specificity.

Next, for the significance assessment of differential co-expression, we calculate the nominal P-value for each individual gene pair differential co-expression. The incidence of false positives for the total data, referred as 'family wise error rate', would be too high if not for adjusting the individual nominal P-values. This should be treated with multiple hypothesis testing. However, both the ECF method and the DAGA method lack the multiplicity of nominal P-values which was caused by performing statistical tests on many genes in parallel. Therefore, the family-wise error rate may be much larger than the expected significance level. In contrast, we use the West-Young permutation method [27] to address the problem of multiple hypothesis testing, which specifically considers the dependence structure between genes.

Finally, formulations by the SIG method and the ECF method make dual confirmation of identification. In other words, gene A and gene B will not be classified as a differential co-expressed gene pair until each gene could be identified when the other gene is factored into consideration. For the SIG method, the distribution of correlation ratio is gene-specific. As for the ECF method, the ECF-statistic was introduced and ECF-statistic of the two genes in a pair was asymmetric. This is the reasoning for identifying gene pair with dual confirmation. However, the DAGA method only performed gene pair identification based on single confirmation, which may lead to a large number of false positive gene pairs.

### Explanation of gene pair interactions

In addition to advantages in an algorithm feature, the SIG method can extract and provide insightful biological clues for interpreting certain gene's puzzling mechanisms during prostate cancer progression.

The gene pairs exclusively identified by the SIG method unravel mechanisms for *PPARG *function loss during prostate cancer development. *PPARG *encodes a protein in the peroxisome proliferator activating receptor (*PPAR*) family, and is a critical transcription factor to promote adipogenesis. *PPARG *has been reported to repress prostate cancer under some undetermined mechanisms [28], mainly due to the disadvantage made by adipogenesis for a cell to maintain in undifferentiated state. However, *PPARG *has high expression levels in prostate cancer cells, especially for the more undifferentiated stage [28]. Thus, *PPARG *seems to lose its repression role in prostate cancer. Genetic causes have been excluded since neither mutations in *PPARG *gene nor deletions in chromosomal region were detected in prostate cell lines or in tumours [28]. Therefore, we suppose the inactivation of *PPARG *occurs as a result of gene deregulation. Since *PPARD *has been suggested as a potent inhibitor for transcription activity of *PPARG *[29] based on the gene pairs exclusively detected by the SIG method, we provide explicable clues of *PPARD*'s regulation role to promote *PPARG *inactivation.

*PPARD *belongs to *PPAR *family just as *PPARG*. It shows contradictory effects of roles during cellular development. A recent report [29] demonstrated it as rescuing prostate epithelial cells from growth inhibition. In the results of the SIG method, gene pair *PPARD *and *DVL1 *has highly positive correlation at HR stage and little correlation at HS stage. *DVL1 *encodes a protein in Wnt signaling which can inactivate *GSK3B*, prevent it from phosphorylating β-catenin (*CTNNB1*) with subsequent degradation. Because *PPARD *could play a role through activation of downstream genes [30], we assume that *PPARD *may enhance *DVL1*'s transcription in HR stage. Consequently, *GSK3B *is inactivated and the degradation for β-catenin is decreased. Since *CTNNB1 *may repress *CEBPD*'s activity, the latter has effect on enhancing *PPARG*'s activity [31]. Therefore, we conclude that *PPARD*'s activation on *DVL1 *leads to repressing *PPARG*'s activity, and this gives clues for tumour repressor *PPARG*'s inactivation and leads to cancer progression into the HR stage.

Similarly, *PPARD *and *NCOA2 *are exclusively detected by SIG method, with highly negative correlation at HR stage, and little correlation in HS stage. The protein encoded by *NCOA2 *has intrinsic histone acetyltransferase activity, and is required for the maximal *PPARG *activity [31]. Inferring that *PPARD *also represses *NCOA2*'s activity, provides another clue for *PPARD*'s inactivation in prostate cancer progression.

### Construction of gene interaction network

An important characteristic of the SIG method is that from its ability to identify differentially co-expressed gene pairs, we can track cancer progression at the molecular level, and obtain some biochemical information about 'evolution footprints'. In order to comprehend prostate cancer progression mechanisms, we exemplify prostate cancer progression from HS stage to HR stage. Deprived of androgen, cells at HS stage are confronted with survival selection. Despite of the neutral changes of gene pair co-regulation, under the pressure of androgen ablation, many cancer cells will die. Some malignant cells survive by developing proper anti-apoptosis strategies and finally adapt to hormone-refractory environment. Eventually the entire tumour becomes hormone unresponsive and loses growth control.

**Figure 4:**
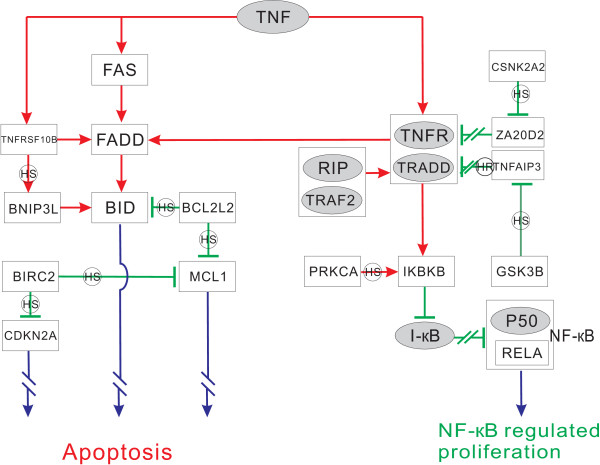
**TNF-induced NF-κB and apoptosis pathways**. Gene in a dark ellipse means it displays differential effects but is missed in the microarray data set. A red arrow indicates an inferred positive effect, and a green bar indicates an inferred negative effect. Double slashes means the effect is blocked. A rectangle containing two genes indicates that these two proteins constitute a complex. If a gene-to-gene effect is inferred to only occur in HS stage or HR stage, the line between the two genes is marked with 'HS' or 'HR' in a small circle, respectively. A blue arrow suggests a lead to final effect, such as apoptosis or proliferation. Note that p50 and RELA constitute the complex NF-κB. This figure illustrates the completions between NF-κB pathway and apoptosis pathway represented by molecular regulation changes during cancer progression.

During this progression from HS stage to HR stage, the TNF signalling pathway plays an important role. It has been reported that in the early cancer stage, TNF plays the role as tumour repressor, but in the late cancer stage, it promotes cell proliferation [32]. The underlying mechanism of this process is still unclear. A network integrating TNF-induced NF-κB pathway and TNF-induced apoptosis pathway together, is inferred built on the gene pairs identified by the SIG method (illustrated in Figure 4). The NF-κB pathway is well known for cell proliferation and for direct activation of androgen receptor [33]. This network reveals the competitive relationship between the two pathways, and indicates the mechanisms of the function alteration of TNF during prostate cancer progression. The details of biological inference for gene pairs and network construction can be consulted in additional file 3.

To achieve NF-κB activation, TNF induces the activation of *IKBKB*, leading to proteolytic degradation of I-κB; then NF-κB is liberated from I-κB's control, allowing NF-κB to translocate into nuclear, to regulate the transcription of various downstream genes involved in carcinogenesis to suppress apoptosis. TNF-induced activation of *IKBKB *first requires TNF to stimulate *TNFR1*, then the ligand-binded *TNFR1 *can recruit *TRADD*, in order to serve together as an assembly platform for binding *TRAF2 *and *RIP*. *TRAF2 *is sufficient to recruit *IKBKB *into *TNFR1 *complex whereas *RIP *is necessary for *IKBKB *activation [34]. In apoptosis pathway, *TNFR1 *also acts as a death receptor. Other death receptors such as *FAS *and *TNFRSF10B *directly bind to *FADD *to initiate apoptosis, whereas *TNFR1 *interacts indirectly with *FADD *through *TRADD*, which is also responsible for bridging *TNFR1 *to *TRAF2 *in NF-κB activation. Therefore, both pathways of NF-κB and apoptosis were competing for *TNFR1*, and the self-inhibitory circuits determine this predominance. Self-inhibitory circuit refers to the termination of TNF-induced response, in which *ZA20D2 *and *TNFAIP3 *play the key roles. *TNFAIP3 *can inhibit both NF-κB activation and *TNFR1*-mediated apoptosis, whereas *ZA20D2 *only inhibits NF-κB activation [35]. In our identified gene pairs, *ZA20D2 *shows negative correlation with *CSNK2A2 *only at HR stage but little correlation at HS stage, and is inferred to be degraded by *CSNK2A2*. This implies that at HR stage, the self-inhibitory circuit for NF-κB signalling has been blocked, and therefore NF-κB prevails in the competition for *TNFR1*. Besides, *TNFAIP3 *shows strong negative correlation with *GSK3B *at HS stage but weak correlation at HR stage, and thus is inferred to be degraded by *GSK3B*. At HS stage, apoptosis was not initiated since cells were not deprived of androgens, so in the competition for TNF's participation in NF-κB activation and apoptosis, NF-κB took an advantageous place. However, based on results derived from the SIG method, we find NF-κB would be finally inactivated by *PPP3R1*'s dephosphorylation on *RELA*. This attenuates cancer aggravation at HS stage. In addition, as illustrated in Figure 4, at HR stage, the already initiated apoptosis caused by androgen ablation may be blocked in various schemes for cell survival.

**Figure 5:**
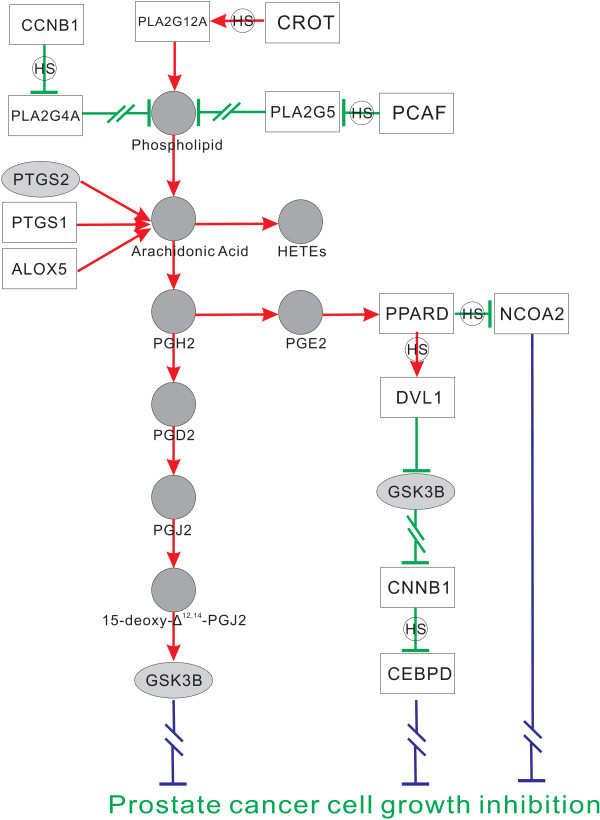
**Arachidonic acid metabolism pathway**. The annotations of symbols and lines are the same as Figure 4. This figure shows the molecular signs represented by gene pair co-expression changes for arachidonic acid (AA) metabolism and prostanglandin generation in promoting prostate cancer progression.

**Figure 6:**
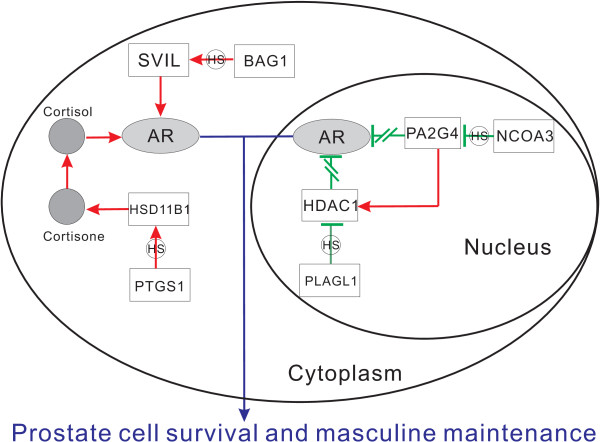
**Androgen receptor pathway**. The annotations of symbols and lines are the same as Figure 4. This figure suggests at HR stage, the aberrances in AR signalling can be manifested in two steps. The first step is the activation of AR including its transport into nucleus without androgen induction, and the second step is the regulation of nuclear coactivators and repressors for AR's transactivation.

In addition, there are also some other pathways have been inferred, such as the arachidonic acid metabolism pathway (illustrated in Figure 5) and androgen receptor signalling pathway (illustrated in Figure 6), which are also very critical in prostate cancer progression. These pathways are presented in details in additional file 3.

## Conclusion

We presented a novel method named the SIG to identify gene pairs with significant differential co-expression patterns in two cellular states with progression relationship. For the first time, the SIG method combines gene co-expression pattern change study with the concept of dynamic cancer progression. This was formulated by applying a stochastic process model to approximate gene co-expression change procedure during cancer progression. This method was applied to two prostate cancer data sets and systematically compared the results with other two current similar methodologies. The results show a high reliability in identifying gene pairs relevant to cancer progression, in gene pair ontology analysis, and in pathway enrichment analysis. Therefore, this method provides insights into understanding carcinogenesis by appropriately tracking major molecular mechanisms of cancer progression, and to serve as a new tool for future cancer etiology study. The SIG method is available as a free software source at .

## Methods

### Overview of SIG method

We present the SIG method based on stochastic process model, to examine genome-wide expression profiles from cancer samples at two stages correlated with progression from stage 1 (early stage) to stage 2 (late stage). The SIG method aims to find gene pairs with significant co-expression change between two stages, i.e. gene pairs have high co-expression in one cancer stage, and have little correlation in the other cancer stage.

There are three key steps in the SIG method:

Step 1: Calculating a correlation ratio (CR) to evaluate gene pair co-expression change. We calculate a ratio of *z*-transformed correlation that reflects the grade to which a gene pair is differentially co-expressed between two cancer stages.

Step 2: Estimating the significance level of CR. For a gene in interest, we estimate the statistical significance (nominal P-value) of the CR formed by another gene with it. Based on a random walk model in stochastic process, we construct an analytical distribution of CRs formed by all other genes with the gene factored into consideration to work as a background for significance assessment. This analytical distribution preserves gene specificity and cancer progression pertinence to better fit complex correlation structure in gene expression data.

Step 3: Adjusting for multiple hypothesis testing. When an entire database of genes is evaluated, to reduce the family wise error we adjust the estimated significance to account for multiple hypothesis testing by using Westfall-Young permutation, which exactly takes advantage of the dependence structure between genes.

### Random walk model

The random walk model is a simple case of stochastic process. The basic idea of a random walk can be described by a drunkard's tottering along a street in a stepwise process: he may walk forward or backward stumblingly with the same probability at each step as generally assumed. This drunkard is known to initially depart from the zero position, and the probability of his being at the position *x *after time *τ *passed is modelled as follows

(4)

where  is the probability. After differential approximation, the solution of Equation (4) is approached as:

(5)

where *u *is the probability density as  and *D *is a constant as . This solution represents the cumulative effect of many random events over the time interval *τ*. It is the general premise that for a cancer cell at each mutation step, the co-expression of a gene pair varies randomly with the same chance to increase or decrease. Therefore, it is very similar to the random walk carried out in a stochastic form.

### Stochastic process-based analytical distribution of correlation ratio

For a gene pair (A, B), we use Pearson correlation coefficient (CC) denoted as *r *to measure their co-expression. *r *is calculated in both stage 1 and stage 2. Then we transform *r *into a metric *z *using Fisher's *r*-to-*z *transformation as z = ln[(1 + *r*)/(1 - *r*)]/2. After the transformation, *z *is well known to be normally distributed [36]. We use the *z*-transformed correlation coefficient (*z*-CC) to measure the co-expression of a gene pair, and in the following text, we name *z*-CC as 'correlation' for simplicity.

For a gene A of interest, let *X *denote the correlations of all other genes with it in one stage, and *Y *denote the corresponding correlations in the other stage. Since correlation has been *z*-transformed, both *X *and *Y *are normally distributed:

(6)

and

(7)

where *x *and *y *are the values of correlation, *μ*_*X*_, *μ*_*Y *_and *σ*_*X*_, *σ*_*Y *_are the expectations and standard deviations of *X *and *Y *respectively, which are be estimated using standard methods.

We use the ratio of correlation of one stage relative to the other stage for measuring the co-expression change of a gene pair. The ratio of correlation is denoted as *T*, and calculated as *T *= *X*/*Y*. The analytical distribution of the ratio of correlation is described as follows:

(8)

where *t *is the value of variant *T*, *f*_*XY*_(*x*, *y*) is the joint probability density of *X *and *Y*. The co-expression of a gene pair progresses from an initial value in the early stage to a final value in the late stage, thus *X *and *Y *are dependent on each other. The joint probability of *X *and *Y *should be calculated using the conditional probability, which measures the probability of a correlation transformed to a final value when its initial value is known. Due to the direction that cancer only progressing from stage 1 to stage 2, there are two cases when computing the conditional probability of *X *and *Y *during the calculation of analytical distribution.

1) *X *belongs to stage 2, and *Y *belongs to stage 1

In this case, the joint probability of a gene pair's correlation values in two cancer stages is represented as:

(9)

where *G*(*x*|*y*) is the conditional probability density for a correlation transforming from value *y *in stage 1 into value *x *in stage 2. For this point, the conditional probability acts as the keystone in our theory. Since the conditional probability is highly correlated with progression between the two stages. Herein, we combine the differential co-expression study with stochastic process model by using the solution of random walk model to approximate the condition probability of correlations. According to random walk model, for a prostate cancer cell, the correlation of a gene pair has equal chance to increase or decrease, therefore *G*(*x*|*y*) has similar cumulative effect with  as shown in Equation (4), and is approximated in the following formula. Please refer to additional file 3 for the detailed derivation.

(10)

where *d *represents the progression distance from stage 1 to stage 2, and *D *is a constant as defined in Equation (5). For generality, we set *d *= 1 and *D *= 1 for the investigations in this paper. The joint probability in Equation (9) is computed as:

(11)

Note that *x *= *ty*, then by combining Equations (8) and (11), the analytical distribution function for the ratio of correlation of stage 2 relative to stage 1 is presented as:

(12)

where , , , , and *K *is a normalization constant.

**Figure 7:**
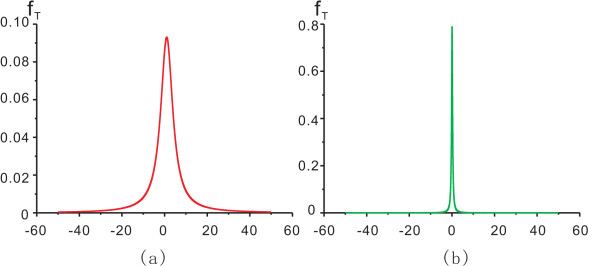
**Analytical distribution function of *z*-CC ratio between HS stage and HR stage**. (a) The analytical distribution *f*_T _of *z*-CC ratio *T *= *X*/*Y*, *X *represents *z*-CCs at the HR stage and Y denotes z-CCs at the HS stage. *T *represents the gene pair co-expression alteration. (b) The theoretical distribution *f*_T _of *z*-CC ratio *T *= *X*/*Y*, *X *and *Y *represent z-CCs at HS stage and at HR stage respectively.

A typical curve of *f*_*T*_(*t*) is plotted in Figure 7a, using the prostate cancer data of HS samples versus HR samples [20] as example. The profile of *f*_*T*_(*t*) is semblable to that of normal distribution, but *f*_*T*_(*t*) has higher density in the center and lower density in the tails. For a given gene, its analytical distribution of the ratio of correlations formed by all other genes pairing this gene, is treated as a background to assess the significance of the observed values of the ratio of correlations. Based on the analytical distribution, gene pairs with absolutely large values of correlation ratio could be identified, i.e. gene pairs with high co-expression in stage 2 and little correlation in stage 1 are regarded as significantly differentially co-expressed.

It should be noted that there is a limitation in our methodology. If the correlation changes from positive to negative or from negative to positive, the corresponding ratio (referred as "P/N ratio") is negative and most of them are not far away from 0. If P/N ratio is negatively large enough, then the corresponding gene pair could be identified. However, there is still a small part of P/N ratios escaping from being identified, and these gene pairs would be missed accordingly.

2) *X *belongs to stage 1, and *Y *belongs to stage 2

In this case, the joint probability for a gene pair's correlation values in two cancer stages is represented as:

(13)

where *G*(*y*|*x*) is the conditional probability density for the correlation transforming from an initial value *x *in stage 1 into a final value *y *in stage 2. After similar derivations, the analytical distribution function *f*_*T*_(*t*) for the correlation ratio of stage 1 relative to stage 2 is given as

(14)

where , , , , and *K*' is a normalization constant.

There are some essential differences between Equations (12) and (14), mainly manifested by the expressions of *a *vs. *a*', and *b *vs. *b*'. Detailed derivations for Equations (12) and (14) are demonstrated in the additional file 3.

A typical curve for the analytical distribution *f*_*T*_(*t*) of correlation ratio in Equation (14) is plotted in Figure 7b, also based on the data set of HS vs. HR. Notice that most correlation ratios (stage 1/stage 2) are very close to zero; whereas in Figure 7a, correlation ratios (stage 2/stage 1) scatter more incompactly from 0. This suggests that gene pairs may have more co-expressions in stage 2 (HR) than in stage 1 (HS); in other words, it implies that the number of gene-gene interactions, either activation or repression, increases remarkably during prostate cancer evolution.

### Estimating significance of gene pair differential co-expression

For a gene of interest, since the correlation ratio (CR) is calculated as a quantification of evidence for differential co-expression, a cut-off for identification is needed by using a false discovery rate criterion, or in other words, the significance level. This process involves calculating the null hypothesis distribution of CR that contains little co-expression change. The profile character of the analytical distribution function *f*_*T*_(*t*) has the properties similar to null hypothesis, and also keeps information about gene specificity. In our methodology, the calculation of null distribution is achieved through an approximation of the analytical distribution of the interested gene. Importantly, this approximation preserves gene specificity and progression character, in order to make gene pair identification without systematic derivation. Therefore, it provides a more biologically reasonable assessment of significance than that obtained by the traditional simulation data.

Therefore, we calculate the nominal P-value of a correlation ratio for significance estimation, based on the analytical distribution *f*_*T*_(*t*) of the gene in interest. The calculation is carried out by the area integral of distribution function.

By setting a false discovery rate *α *to the nominal P-value, in principle, gene pair co-expression change can be determined as whether it is significant or not. However, the incidence of false positives for the total data, referred as 'family wise error rate', would be high if we do not adjust individual nominal P-values. We use the Westfall-Young Permutation [27] to do the multiple hypothesis testing to correct for false positive occurrence, because this is the only correction for taking advantage of dependence structure between genes, and accounts for gene co-regulation (review [37]). In the SIG method, for a given gene, Westfall-Young permutation is employed to calculate adjusted P-value for the pair formed by another gene with the given gene. The details of this multiple hypothesis testing are demonstrated in additional file 3.

### Identifying gene pair with differential co-expression pattern

We propose the following procedure for identifying differential gene pair co-expression patterns in different biological stages. For a gene A, this procedure screens all the other genes B and selects genes that form differentially co-expressed pair with it. For a gene pair (A, B), two analytical distributions  and  may be not the same. To achieve consistency, a pair of genes will be considered to be detected only if their CR-based adjusted P-values in each analytical distributions are both greater than a threshold value *α*.

Taking together, the procedure of determining a gene pair (A, B) with significant co-expression pattern change is described as follows:

1) Calculate the correlation ratio *t*_*AB *_for gene pair (A, B).

2) Figure out the analytical distributions  and .

3) For gene A chosen, calculate the adjusted P-value p* for *t*_*AB*_, if p* <*α*, regard gene B as differentially co-expressed with gene A.

4) For gene B, repeat step 3).

5) If gene A and gene B both have differential co-expression with each other, identify them as a significant differentially co-expressed pair.

In this paper, we set P-value threshold *α *= 0.05.

## List of abbreviations

SIG: Stochastic process model for Identifying differentially co-expressed Gene pair; HS: Hormone Sensitive; HR: Hormone Resistant; RS: Re-identification Scale; PS: Progression Score; TP: True Positive; FP: False Positive; ND: Non-Discriminatory; TPR: True Positive Rate; PES: Pathway Enrichment Score; CC: Correlation Coefficient; *z*-CC: *z*-transformed Correlation Coefficient; CR: Correlation Ratio; Avg: Average.

## Authors' contributions

WM planed and designed the method, carried out the data analysis, performed the further analysis in biological functions, drafted the manuscript, and mainly generated the figures. XF organized this research, co-worked in the method design and data analysis, as well as manuscript typewriting. FG wrote the code program. XH, GY, YH, JZ and YM also performed the data analysis and contributed in editing the manuscript. YL and YX organized all the research and provided advice for preparing the manuscript. All authors read and approved the final manuscript.

## Supplementary Material

Additional file 1 **Gene pairs mentioned in biological analysis**. In this supplementary file, gene pairs mentioned in the biological interpretation and network inference sections are listed, with differential co-expression patterns between the HS stage and the HR stage built on the SIG method. In addition, correlation coefficient and Progression Score (PS) are listed. Click here for file

Additional file 2 **PES comparison for three methods based two data sets**. In this supplementary file, based on two data sets (Best CJ et al.'s prostate cancer data for HS samples versus HR samples; Singh D et al.'s prostate data for healthy samples versus cancerous samples), we list the Pair Enrichment Score (PES) results derived by three methods (the SIG method, the ECF method, and the DAGA method) for 144 curated pathways. Click here for file

Additional file 3 **SIG algorithm & Biological inference**. In this supplementary file, we give the detailed description of the SIG algorithm, and also provide the detailed biological inference for networks. Click here for file

## References

[B1] DeRisi JL, Iyer VR, Brown PO (1997). Exploring the metabolic and genetic control of gene expression on a genomic scale. Science.

[B2] Grigoriev A (2001). A relationship between gene expression and protein interactions on the proteome scale: analysis of the bacteriophage T7 and the yeast Saccharomyces cerevisiae. Nucleic Acids Res.

[B3] Jansen R, Greenbaum D, Gerstein M (2002). Relating whole-genome expression data with protein-protein interactions. Genome Res.

[B4] Ge H, Liu Z, Church GM, Vidal M (2001). Correlation between transcriptome and interactome mapping data from Saccharomyces cerevisiae. Nat Genet.

[B5] Kemmeren P, van Berkum NL, Vilo J, Bijma T, Donders R, Brazma A, Holstege FC (2002). Protein interaction verification and functional annotation by integrated analysis of genome-scale data. Mol Cell.

[B6] Lai Y (2008). Genome-wide co-expression based prediction of differential expressions. Bioinformatics.

[B7] Lai Y, Wu B, Chen L, Zhao H (2004). A statistical method for identifying differential gene-gene co-expression patterns. Bioinformatics.

[B8] Choi JK, Yu U, Yoo OL, Kim S (2005). Differential coexpression analysis using microarray data and its application to human cancer. Bioinformatics.

[B9] Yoon SH, Kim JS, Song HH (2003). Statistical inference methods for detecting altered gene associations. Genome Inform.

[B10] Li KC (2002). Genome-wide coexpression dynamics: theory and application. Proc Natl Acad Sci USA.

[B11] Komarova NL, Sengupta A, Nowak MA (2003). Mutation-selection networks of cancer initiation: tumor suppressor genes and chromosomal instability. Journal of Theoretical Biology.

[B12] Speer JF, Petrosky VE, Retsky MW, Wardwell RH (1984). A stochastic numerical model of breast cancer growth that simulates clinical data. Cancer Res.

[B13] Little MP, Wright EG (2003). A stochastic carcinogenesis model incorporating genomic instability fitted to colon cancer data. Math Biosci.

[B14] Conolly RB, Kimbell JS (1994). Computer Simulation of Cell Growth Governed by Stochastic Processes: Application to Clonal Growth Cancer Models. Toxicology and Applied Pharmacology.

[B15] Kimmel M, Axelrod DE (1990). Mathematical models of gene amplification with applicationst to cellular drug resistance and tumorigenicity. Genetics.

[B16] Paxia S, Rudra A, Zhou Y, Mishra B (2002). A random walk down the genomes: DNA evolution in Valis. Computer.

[B17] Yokota H, Engh G van den, Hearst JE, Sachs RK, Trask BJ (1995). Evidence for the organization of chromatin in megabase pair-sized loops arranged along a random walk path in the human G0/G1 interphase nucleus. J Cell Biol.

[B18] Landis SH, Murray T, Bolden S, Wingo PA (1999). Cancer statistics. CA Cancer J Clin.

[B19] Nelson WG, De Marzo AM, Isaacs WB (2003). Prostate cancer. N Engl J Med.

[B20] Best CJ, Gillespie JW, Yi Y, Chandramouli GV, Perlmutter MA, Gathright Y, Erickson HS, Georgevich L, Tangrea MA, Duray PH (2005). Molecular alterations in primary prostate cancer after androgen ablation therapy. Clin Cancer Res.

[B21] Singh D, Febbo PG, Ross K, Jackson DG, Manola J, Ladd C, Tamayo P, Renshaw AA, D'Amico AV, Richie JP (2002). Gene expression correlates of clinical prostate cancer behavior. Cancer Cell.

[B22] Ross SE, Hemati N, Longo KA, Bennett CN, Lucas PC, Erickson RL, MacDougald OA (2000). Inhibition of adipogenesis by Wnt signaling. Science.

[B23] Chinnaiyan AM, O'Rourke K, Tewari M, Dixit VM (1995). FADD, a novel death domain-containing protein, interacts with the death domain of Fas and initiates apoptosis. Cell.

[B24] Denisov AY, Chen G, Sprules T, Moldoveanu T, Beauparlant P, Gehring K (2006). Structural model of the BCL-w-BID peptide complex and its interactions with phospholipid micelles. Biochemistry.

[B25] Golub TR, Slonim DK, Tamayo P, Huard C, Gaasenbeek M, Mesirov JP, Coller H, Loh ML, Downing JR, Caligiuri MA (1999). Molecular classification of cancer: class discovery and class prediction by gene expression monitoring. Science.

[B26] Subramanian A, Tamayo P, Mootha VK, Mukherjee S, Ebert BL, Gillette MA, Paulovich A, Pomeroy SL, Golub TR, Lander ES (2005). Gene set enrichment analysis: a knowledge-based approach for interpreting genome-wide expression profiles. Proc Natl Acad Sci USA.

[B27] Westfall PH, Young SS (1993). Resampling-based Multiple Testing.

[B28] Mueller E, Smith M, Sarraf P, Kroll T, Aiyer A, Kaufman DS, Oh W, Demetri G, Figg WD, Zhou XP (2000). Effects of ligand activation of peroxisome proliferator-activated receptor gamma in human prostate cancer. Proc Natl Acad Sci USA.

[B29] Jarvis MC, Gray TJB, Palmer CNA (2005). Both PPARgamma and PPARdelta influence sulindac sulfide-mediated p21WAF1/CIP1 upregulation in a human pnrostate epithelial cell line. Oncogene.

[B30] Suchanek KM, May FJ, Lee WJ, Holman NA, Roberts-Thomson SJ (2002). Peroxisome proliferator-activated receptor beta expression in human breast epithelial cell lines of tumorigenic and non-tumorigenic origin. Int J Biochem Cell Biol.

[B31] Rosen ED, Walkey CJ, Puigserver P, Spiegelman BM (2000). Transcriptional regulation of adipogenesis. Genes Dev.

[B32] Balkwill F (2002). Tumor necrosis factor or tumor promoting factor?. Cytokine Growth Factor Rev.

[B33] Zhang L, Charron M, Wright WW, Chatterjee B, Song CS, Roy AK, Brown TR (2004). Nuclear factor-kappaB activates transcription of the androgen receptor gene in Sertoli cells isolated from testes of adult rats. Endocrinology.

[B34] Devin A, Cook A, Lin Y, Rodriguez Y, Kelliher M, Liu Z (2000). The distinct roles of TRAF2 and RIP in IKK activation by TNF-R1: TRAF2 recruits IKK to TNF-R1 while RIP mediates IKK activation. Immunity.

[B35] He KL, Ting AT (2002). A20 inhibits tumor necrosis factor (TNF) alpha-induced apoptosis by disrupting recruitment of TRADD and RIP to the TNF receptor 1 complex in Jurkat T cells. Mol Cell Biol.

[B36] Snedecor GW, Cochran WG (1980). Statistical Methods.

[B37] Dudoit S, Yang Y, Callow M, Speed T (2002). Statistical methods for identifying differentially expressed genes in replicated cDNA microarray experiments. Statistica Sinica.

