# Supplementary material for: A stochastic model for identifying differential gene pair co-expression patterns in prostate cancer progression
Source: BMC Genomics. 2009 Jul 29;10:340. doi: 10.1186/1471-2164-10-340 (PMC2737000; doi:10.1186/1471-2164-10-340)
Supplement: Additional file 1 — Gene pairs mentioned in biological analysis. In this supplementary file, gene pairs mentioned in the biological interpretation and network inference sections are listed, with differential co-expression patterns between the HS stage and the HR stage built on the SIG method. In addition, correlation coefficient and Progression Score (PS) are listed. [file 1471-2164-10-340-S1.doc]

**Supplement 1**

**Gene-pairs mentioned in biological analysis**

Gene pairs with differential co-expressions between prostate cancer HS stage and HR stage mentioned in progression analysis, biological interpretation, and network inference, are exclusively identified by the SIG method.

| Function | Gene A | Gene B | r|HS | r|HR | PS |
| --- | --- | --- | --- | --- | --- |
| Arachidonic acid release and eicosanoid production | *PLA2G4A* | *CTNNB1* | -0.0038 | -0.5301 | 1.6221 |
|  | *PLA2G5* | *PCAF* | 0.0076 | -0.4797 | 1.5907 |
|  | *PLA2G12A* | *CROT* | 0 | 0.5363 | 1.6842 |
|  | *PTGS1* | *HSD11B1* | 0.0015 | 0.4027 | 1.6003 |
|  | *PTGS1* | *ALOX5* | -0.0192 | 0.6789 | 1.6291 |
| PPAR family members’ regulation | *PPARD* | *NCOA2* | -0.0192 | -0.6457 | 1.8104 |
|  | *PPARD* | *DVL1* | 0.0003 | 0.5618 | 1.7965 |
|  | *CEBPD* | *CTNNB1* | -0.0070 | -0.6033 | 1.6536 |
| TNF-induced NF-κB activation | *IKBKB* | *PRKCA* | -0.0047 | 0.7040 | 1.7404 |
|  | *RELA* | *PPP3R1* | -0.4975 | -0.0032 | 1.6601 |
|  | *ZA20D2* | *CSNK2A2* | 0.0087 | -0.5427 | 1.7847 |
| TNF-induced apoptosis | *FAS* | *FADD* | 0.0135 | 0.4859 | 1.8055 |
|  | *TNFRSF10B* | *BNIP3L* | 0.0127 | 0.5732 | 1.5633 |
|  | *TNFAIP3* | *GSK3B* | -0.6698 | 0.0082 | 1.7332 |
| Apoptosis arrest | *BCL2L2* | *BID* | 0.0055 | -0.3084 | 1.8020 |
|  | *BCL2L2* | *MCL1* | -0.0109 | -0.5760 | 1.7044 |
|  | *BIRC2* | *CDKN2A* | -0.0005 | -0.7492 | 1.6756 |
|  | *BIRC2* | *MCL1* | -0.0032 | -0.4410 | 1.7607 |
| Androgen receptor regulation | *BAG1* | *SVIL* | -0.0017 | 0.4024 | 1.6946 |
|  | *HDAC1* | *PLAGL1* | -0.0255 | -0.8818 | 1.7752 |
|  | *NCOA3* | *PA2G4* | -0.0087 | -0.5250 | 1.7278 |

*r*|HS and *r*|HR represent correlation coefficient values of gene pair (*A*, *B*) at prostate cancer HS stage and HR stage, respectively. In addition, a statistic named Progression Score (PS) is calculated for each gene pair to explore its progressive grade.
