# Supplementary material for: A stochastic model for identifying differential gene pair co-expression patterns in prostate cancer progression
Source: BMC Genomics. 2009 Jul 29;10:340. doi: 10.1186/1471-2164-10-340 (PMC2737000; doi:10.1186/1471-2164-10-340)
Supplement: Additional file 3 — SIG algorithm & Biological inference. In this supplementary file, we give the detailed description of the SIG algorithm, and also provide the detailed biological inference for networks. [file 1471-2164-10-340-S3.doc]

**Supplement 3:**

**Part I: SIG algorithm**

In this appendix, we give the detailed descriptions of the SIG algorithm.

For a gene A, let *X* denote the *z*-CCs of all other genes forming with it at HR stage, and *Y* denote the corresponding *z*-CCs at HS stage. The two variants are both normally distributed as

$f_{X}(x)=\frac{1}{\sqrt{2\pi}\sigma_{X}}\exp(-\frac{(x-\mu_{X})^{2}}{2\sigma_{X}^{2}}) ,$ $f_{Y}(y)=\frac{1}{\sqrt{2\pi}\sigma_{Y}}\exp(-\frac{(y-\mu_{Y})^{2}}{2\sigma_{Y}^{2}})$ (1)

where *x* and *y* are the values of *z*-CC, $\mu_{X}$,$\mu_{Y}$ and $\sigma_{X}$,$\sigma_{Y}$ are the expectations and standard deviations respectively, which are estimated using standard methods.

We use the ratio of *z*-CCs of one stage relative to another stage to measure the change of a gene pair co-expression. Set *z*-CC ratio $T=X/Y$, its analytical distribution is:

$f_{T}(t)=\int_{-\infty}^{\infty} f_{XY}(x,y)|y|dy\text{ }=\int_{0}^{\infty} f_{XY}(ty,y)ydy-\int_{-\infty}^{0} f_{XY}(ty,y)ydy$, (2)

where *t* is the value of variant *T*, $f_{XY}(x,y)$ is the joint probability density of *X* and *Y*.

When a cancer progresses from HS stage into HR stage, the probability for a gene pair’s *z*-CC value being *y* at the early cancer stage and being *x* at the late cancer stage can be represented as

$f_{XY}(x,y)=G(x|y)f_{Y}(y)$ (3)

where $G(x|y)$ is the conditional probability density for *z*-CC value transforming from *y* at HS stage into *x* at HR stage. We can derive the conditional probability $G(x|y)$ from the theory of stochastic process.

Generally, there is a simple case of stochastic process – the random walk model. The basic idea of a random walk can be described by a drunkard’s tottering along a street in a stepwise process: he may walk forward or backward stumblingly with the same probability at each step. This drunkard is known to initially depart from the zero position, and the probability of his being at the position x after time $\tau$ passed is modeled as follows

(4)

where $\bar{\omega}(x,\tau)$ is the probability for a particle at position *x* when the time is $\tau$, assumed the particle known initially departed from the zero position. After differential approximation, the solution of Equation (4) can be approached as

$u\approx u_{0}(x,\tau)\equiv(4\pi D\tau)^{-1/2}\exp(-\frac{x^{2}}{4D\tau})$ (5)

where $u=\bar{\omega}/2\Delta x ,$ $D=\lim_{\Delta x\to0,\Delta\tau\to0} (\Delta x)^{2}/2\Delta x$.

It is the general premise that for a cancer cell at each mutation step, the co-expression of a gene pair varies randomly with the same chance to increase or decrease. Therefore, it is very similar to the random walk carried out in a stochastic form. We can approximate $G(x|y)$ as

$G(x|y)\approx(4\pi Dd)^{-1/2}\exp[-\frac{(x-y)^{2}}{4Dd}]$ (6)

In this equation, *d* refers to the progression distance from AD stage to AI stage, and *D* is a constant as defined in Equation (5). The joint probability density in Equation (3) is computed as

$f_{XY}(x,y)=\frac{1}{2\pi\sqrt{2Dd}\sigma_{Y}}\exp\{-[\frac{(x-y)^{2}}{4Dd}+\frac{(y-\mu_{Y})^{2}}{2\sigma_{Y}^{2}}]\}$ (7)

Note that $x=ty$, then we replace *x* with *ty* in Equation (7), and have

$f_{XY}(ty,y)=\frac{1}{2\pi\sqrt{2Dd}\sigma_{Y}}\exp\{-[\frac{y^{2}(t-1)^{2}}{4Dd}+\frac{(y-\mu_{Y})^{2}}{2\sigma_{Y}^{2}}]\}$. (8)

Then substitute Equation (8) into Equation (2), and obtain

$f_{T}(t)=\frac{1}{2\pi\sqrt{2Dd}\sigma_{Y}}(U-V)$, (9)

where

$U=\int_{0}^{\infty} \exp[-\frac{(t-1)^{2}y^{2}}{4Dd}-\frac{(y-\mu_{Y})^{2}}{2\sigma_{Y}^{2}}]ydy$, (10)

$V=\int_{-\infty}^{0} \exp[-\frac{(t-1)^{2}y^{2}}{4Dd}-\frac{(y-\mu_{Y})^{2}}{2\sigma_{Y}^{2}}]ydy$. (11)

Now we perform the integration of *U* in the following:

$U=\int_{0}^{\infty} \exp\{-\{[\frac{(t-1)^{2}}{4Dd}+\frac{1}{2\sigma_{Y}^{2}}]y^{2}-\frac{\mu_{Y}}{\sigma_{Y}^{2}}y+\frac{\mu_{Y}^{2}}{2\sigma_{Y}^{2}}\}\}ydy$. (12)

Let

$$a=\frac{(t-1)^{2}}{4Dd}+\frac{1}{2\sigma_{Y}^{2}} ,$$

$$b=-\frac{\mu_{Y}}{\sigma_{Y}^{2}} ,$$

$$c=\frac{\mu_{Y}^{2}}{2\sigma_{Y}^{2}} ,$$

then Equation (12) can be written as

$U=\int_{0}^{\infty} \exp[-(ay^{2}+by+c)]ydy$ $=\exp(\frac{b^{2}}{4a}-c)\int_{0}^{\infty} \exp[-a(y+\frac{b}{2a})^{2}]ydy$ (13)

Let $y'=y+\frac{b}{2a}$, then $y=y'-\frac{b}{2a}$, $dy=dy'$, the integral in Equation (13) can be calculated as:

$\begin{matrix} \int_{0}^{\infty} \exp[-a(y+\frac{b}{2a})^{2}]ydy=\int_{b/2a}^{\infty} \exp(-ay'^{2})(y'-\frac{b}{2a})dy' \\ \text{ }=\int_{b/2a}^{\infty} y'\exp(-ay'^{2})dy'-\frac{b}{2a}\int_{b/2a}^{\infty} \exp(-ay'^{2})dy'\text{. } \end{matrix}$ (14)

The first term of Equation (14) is

$\int_{b/2a}^{\infty} y'\exp(-ay'^{2})dy'=\text{-}\frac{1}{\text{2a}}\int_{b/2a}^{\infty} d[\exp(-ay'^{2})]=\frac{1}{\text{2a}}\exp(-\frac{b^{2}}{4a})$, (15)

and the integration in the second term of Equation (14) is

$\int_{b/2a}^{\infty} \exp(-ay'^{2})dy'=\int_{b/2a}^{0} \exp(-ay'^{2})dy'+\int_{0}^{\infty} \exp(-ay'^{2})dy'$ . (16)

Equation (15) can be calculated via the traditional transformation from cartesian coordinates to polar coordinates, and we have

$\int_{0}^{\infty} \exp(-ay'^{2})dy'=\frac{1}{2}\sqrt{\frac{\pi}{a}}$. (17)

Equation (16) can be calculated numerically, and we use *A* to denote the numerated value, i.e.

$A=\int_{b/2a}^{0} \exp(-ay^{2})dy$, (18)

then

$\int_{b/2a}^{\infty} \exp(-ay'^{2})dy'=A+\frac{1}{2}\sqrt{\frac{\pi}{a}}$. (19)

Substitute Equations (15) and (19) into Equation (14), we have

$\int_{0}^{\infty} \exp[-a(y+\frac{b}{2a})^{2}]ydy=\frac{1}{\text{2a}}\exp(-\frac{b^{2}}{4a})-\frac{\text{b}}{\text{2a}}(A+\frac{1}{2}\sqrt{\frac{\pi}{a}})$. (20)

Substitute Equation (20) into Equation (13), then obtain the expression of the first term in Equation (9) as

$U=\int_{0}^{\infty} \exp[-(ay^{2}+by+c)]ydy\text{ }=\exp(\frac{b^{2}}{4a}-c)[\frac{1}{\text{2a}}\exp(-\frac{b^{2}}{4a})-\frac{\text{b}}{\text{2a}}(A+\frac{1}{2}\sqrt{\frac{\pi}{a}})]\text{ }$ (21)

Analogously, the second term in Equation (9) can be written as

$V=\int_{-\infty}^{0} \exp\{-\{[\frac{(t-1)^{2}}{4Dd}+\frac{1}{2\sigma_{Y}^{2}}]y^{2}-\frac{\mu_{Y}}{\sigma_{Y}^{2}}y+\frac{\mu_{Y}^{2}}{2\sigma_{Y}^{2}}\}\}ydy$. (22)

Let $y'=-y$, then

$\begin{matrix} V=-\int_{0}^{\infty} \exp\{-\{[\frac{(t-1)^{2}}{4Dd}+\frac{1}{2\sigma_{Y}^{2}}]y'^{2}+\frac{\mu_{Y}}{\sigma_{Y}^{2}}y'+\frac{\mu_{Y}^{2}}{2\sigma_{Y}^{2}}\}\}y'dy' \\ \text{ }=-\int_{0}^{\infty} \exp[-(a'y'^{2}+b'y'+c')]y'dy' \end{matrix}$ (23)

where $a'=a$, $b'=-b$, $c'=-c$. Compared with Equation (21), the expression of *V* can be written as

$V=-\exp(\frac{b^{2}}{4a}-c)[\frac{1}{\text{2a}}\exp(-\frac{b^{2}}{4a})-\frac{\text{b'}}{\text{2a}}(A'+\frac{1}{2}\sqrt{\frac{\pi}{a}})]$ (24)

where

$\begin{matrix} A'=\int_{b'/2a}^{0} \exp(-ay^{2})dy=\int_{-b/2a}^{0} \exp(-ay^{2})dy \\ \text{ }=-\int_{0}^{-b/2a} \exp(-ay^{2})dy=-\int_{b/2a}^{0} \exp(-ay^{2})dy=-A\text{ }. \end{matrix}$ (25)

Then we have

$V=-\exp(\frac{b^{2}}{4a}-c)[\frac{1}{\text{2a}}\exp(-\frac{b^{2}}{4a})+\frac{\text{b}}{\text{2a}}(-A+\frac{1}{2}\sqrt{\frac{\pi}{a}})]$ (26)

Substitute Equations (21) and (26) into Equation (9), we finally obtain the simplified form of $f_{T}(t)$ as

$f_{T}(t)=K\frac{1}{2\pi\sqrt{2Dd}\sigma_{Y}a}\exp(-c)[1-bA\exp(\frac{b^{2}}{4a})]$ (27)

where $a=\frac{(t-1)^{2}}{4Dd}+\frac{1}{2\sigma_{Y}^{2}} ,$ $b=-\frac{\mu_{Y}}{\sigma_{Y}^{2}} ,$ $c=\frac{\mu_{Y}^{2}}{2\sigma_{Y}^{2}} ,$ the integration $A=\int_{b/2a}^{0} \exp(-ay^{2})dy$ can be figured out by numerical calculation, and *K* is a normalization constant to ensure the integration of $f_{T}(t)$ to be 1.

However, according to the *z*-CC ratio distribution described above, only ratios with absolutely large values could be identified as differentially co-expressed, i.e. only gene-pairs showing relatively tight co-expression at HR stage and almost no co-expression at HS stage can be identified. Nevertheless, *t* values of the pairs highly correlated at HS stage and lowly correlated at HR stage would be close to 0. Then, how to find out the pairs showing high co-expression at HS stage but low co-expression at HR stage? Likewise, we also define the *z*-CC ratio as $T=X/Y$, here *X* represents *z*-CCs at HS stage and *Y* denotes *z*-CCs in HR stage. As prostate cancer only progresses from HS stage to HR stage the conditional probability and the distribution of *T* are not entirely the same as inferred before. The derivation is as follows.

Let *X* denote *z*-CC at HS stage, and *Y* denote *z*-CC at HR stage, then

$f_{T}(t)=\int_{-\infty}^{\infty} f_{XY}(x,y)|y|dy\text{ }=\int_{0}^{\infty} f_{XY}(ty,y)ydy-\int_{-\infty}^{0} f_{XY}(ty,y)ydy$. (28)

The evolution of *z*-CC is from *x* to *y*, then the joint probability density $f_{XY}(x,y)$ can be written as

$f_{XY}(x,y)=\frac{1}{2\pi\sqrt{2Dd}\sigma_{X}}\exp\{-[\frac{(y-x)^{2}}{4Dd}+\frac{(x-\mu_{X})^{2}}{2\sigma_{X}^{2}}]\}$. (29)

Replace *x* with *ty* in Equation (29), we have

$f_{XY}(ty,y)=\frac{1}{2\pi\sqrt{2Dd}\sigma_{X}}\exp\{-[\frac{(t-1)^{2}y^{2}}{4Dd}+\frac{(y-\mu_{X}/t)^{2}t^{2}}{2\sigma_{X}^{2}}]\}$. (30)

Note that the form of Equation (30) is different from that of Equation (8), which is ascribed to the progression direction of disease. Substitute Equation (30) into Equation (28), we obtain

$f_{T}(t)=\frac{1}{2\pi\sqrt{2Dd}\sigma_{X}}(U-V)$, (31)

where

$U=\int_{0}^{\infty} \exp[-\frac{(t-1)^{2}y^{2}}{4Dd}-\frac{(y-\mu_{X}/t)^{2}t^{2}}{2\sigma_{X}^{2}}]ydy$, (32)

$V=\int_{-\infty}^{0} \exp[-\frac{(t-1)^{2}y^{2}}{4Dd}-\frac{(y-\mu_{X}/t)^{2}t^{2}}{2\sigma_{X}^{2}}]ydy$. (33)

Let $\mu_{X}'=\mu_{X}/t$, and $\sigma_{X}'=\sigma_{X}/t$, then

$U=\int_{0}^{\infty} \exp[-\frac{(t-1)^{2}y^{2}}{4Dd}-\frac{(y-\mu_{X}')^{2}}{2\sigma'_{X}^{2}}]ydy$, (34)

$V=\int_{-\infty}^{0} \exp[-\frac{(t-1)^{2}y^{2}}{4Dd}-\frac{(y-\mu_{X}')^{2}}{2\sigma'_{X}^{2}}]ydy$, (35)

which have the same form of Equations (10) and (11) respectively. Therefore, the final expression of $f_{T}(t)$ is

$f_{T}(t)=K\frac{1}{2\pi\sqrt{2Dd}\sigma_{X}a}\exp(-c)[1-bA\exp(\frac{b^{2}}{4a})]$, (36)

where

$a=\frac{(t-1)^{2}}{4Dd}+\frac{1}{2\sigma'_{X}^{2}}=\frac{(t-1)^{2}}{4Dd}+\frac{t^{2}}{2\sigma_{X}^{2}}$,

$b=-\frac{\mu_{X}'}{\sigma'_{X}^{2}}=-\frac{t\mu_{X}}{\sigma_{X}^{2}}$, $c=\frac{\mu'_{X}^{2}}{2\sigma'_{X}^{2}}=\frac{\mu_{X}^{2}}{2\sigma_{X}^{2}}$, $A=\int_{b/2a}^{0} \exp(-ay^{2})dy$,

and *K* is a normalization constant.

## Multiple hypothesis testing

Suppose in the original data matrix, there are *n* columns of samples, and *k*+1 rows of genes, i.e. for a gene factored into consideration, the other *k* genes forming pairs with it. The correction procedure is described as follows.

1. To this gene, for each pair formed by another gene with it, calculate the correlation ratio CR, and estimate nominal p values according to its analytical distribution $f_{T}(t)$, based upon the original data. Then rank the p values in an ascending order, i.e. $P_{r_{1}}\leq P_{r_{2}}\leq\ldots\leq P_{r_{k}}$, and $r_{k}$ is especially referred to the original data.
2. For the *b*th permutation
3. Permute the *n* columns of original data matrix, except for the fixed gene.
4. For each gene ranked in the $r_{j}$ order, compute their correlation ratios with the fixed gene, and calculate the corresponding nominal p values according to the original distribution function $f_{T}(t)$ of the given gene.
5. Compute the new p values, referred as *u*, in a step-down process

$u_{k}^{(b)}=p_{r_{k}}^{(b)}$ ,

$u_{j}^{(b)}=\min(u_{j+1}^{(b)},p_{r_{j}}^{(b)})\text{ , for }1\leq j\leq k-1$,

1. Repeat step 2) B times, and for each gene $r_{j}$, the adjusted p value is estimated by

$\tilde{p}_{r_{j}}^{*}=\frac{\sum_{b=1}^{B} I(u_{j}^{(b)}\leq p_{r_{j}})}{B},$ (37)

where $I(\cdot)$ is the indicator function equal to 1 if the condition in parentheses is true, and 0 otherwise. For the examples in this study, we set the permutation number *B*=10^4^.

1. If $\tilde{p}_{r_{j}}^{*}<\alpha$, the pair formed by this corresponding gene $r_{j}$ with the fixed gene is regarded as possessing significant co-expression change between different biological conditions. Here $\alpha$ refers to a predefined significance threshold value.

**PES calculation**:

For a known gene pair set *S* with $N_{H}$ possible gene pairs, the procedure for calculating its PES value is presented in the following.

i) Rank descending order the *N* identified gene pairs to form list $L=\{g_{1},\cdots,g_{N}\}$ according to the correlation of their co-expression change with the biological condition distinction by using any suitable metric$r(g_{j})=r_{j}$. For example, in the SAP method, correlation ratio is taken into account as the metric for co-expression change.

ii) The score is calculated by walking down the list *L*, increasing a running-sum statistic when we encounter a gene pair in S (“hits”) and decreasing it when we encounter gene pairs not in S (“misses”). The magnitude of the increment depends on the correlation of the gene pair co-expression change with the phenotype distinction. We evaluate the fraction of identified gene pairs in *S* and the fraction of gene pairs not in *S* present up to a given position *i* in *L*.

$P_{hit}(S,i)=\sum_{\begin{matrix} g_{j}\in S \\ j\leq i \end{matrix}} \frac{|r_{j}|^{P}}{N_{R}}$, $P_{miss}(S,i)=\sum_{\begin{matrix} g_{j}\in S \\ j\leq i \end{matrix}} \frac{1}{N-N_{H}}$,

where $N_{R}=\sum_{g_{j}\in S} |r_{j}|^{P}$, *p* is the enrichment weighting exponent. We set *p* = 1 in the examples, which weighs the gene pairs in *S* by their correlation with biological condition distinction, normalized by the sum of the correlations over all of the gene pairs in *S*. The PES is the maximum deviation from zero of $|P_{hit}-P_{miss}|$. For a randomly distributed *S*, PES(*S*) will be small; but if *S* is concentrated at the top or bottom of the list, or otherwise nonrandomly distributed, then PES(*S*) will be high.

For the SIG method, correlation ratio is the metric for gene pair co-expression change, and according to which we merge the total results of two cases into one rank. For Lai YL *et al.*’s ECF method, the ECF statistic is the metric, and directly used to rank the identified pairs. For Yoon SH *et al.*’s DAGA method, correlation ratio is the metric for co-expression change.

## Statistical power calculation

In addition to the SIG method, we calculate its power in the following. Power of a [statistical test](http://en.wikipedia.org/wiki/Statistical_hypothesis_testing) is the probability that the test will reject a false [null hypothesis](http://en.wikipedia.org/wiki/Null_hypothesis) (that it will not make a [Type II error](http://en.wikipedia.org/wiki/Type_I_and_type_II_errors)), it reflects the ability of a test to detect an effect, given that the effect actually exists. The probability of a Type II error is referred to as the [false negative rate](http://en.wikipedia.org/wiki/Type_I_and_type_II_errors#False_negative_rate) (β). Since statistical power = 1-β, as power increases, the chances of a Type II error decrease. In a statistical test, when we control the type I error, referred to as ‘significance level’), we also wish the probability of type II error is small. Here, to an observed CR value for a fixed gene, we provide the formulas to calculate [false negative rate](http://en.wikipedia.org/wiki/Type_I_and_type_II_errors#False_negative_rate)βbased on the null hypothesis distribution approximated by analytical distribution of CR.

Set a threshold $\alpha$ for the significance level. For the negative portion of analytical distribution, denote the left cut off as *T*_1_; and for the positive portion, denote the right cut off as *T*_2_. We calculate βfor an observed CR value (denoted as ‘*T*’) only when its corresponding nominal p value is smaller than threshold $\alpha$. There are two cases.

1. When $T<T_{1}$, define $d=T_{1}-T$, then

$\beta=\int_{1+d}^{+\infty} f_{T}(t)dt$. (38)

1. When $T>T_{2}$, define $d=T-T_{2}$, then

$\beta=\int_{-\infty}^{1-d} f_{T}(t)dt$. (39)

## Expression data requirements

Based on the SIG method, there are several requirements for the expression data used.

(1) For correlation coefficient calculation, at least six samples are required at each cancer stage.

(2) Since we examine the correlation change for a gene pair, the sample numbers for two cancer stages should be more or less coincident.

**Part II: Biological interpretations and network inferences**

**Arachidonic acid metabolism pathway.**

Since a decade ago, a number of epidemiologic studies have suggested that high consumption of fat, especially red meat, is a risk factor for prostate cancer (Norrish, 1999; *etc.*). In our results, we focus on the arachidonic acid (AA) metabolism in combination with its downstream substrate *PPAR*s regulation in adipogenesis to investigate lipid’s influence on prostate cancer progression.

In our results, gene pair *PLA2G4A* and *CTNNB1* has differential co-expression pattern, the coefficient is -0.5301 at HR stage and -0.0038 at HS stage. The protein encoded by *PLA2G4A* belongs to the cytosolic phospholipase A2 family, which can mobilize arachidonic acid (AA) from cellular membrane glycerolipid pools into cytoplasm. β-catenin encoded by *CTNNB1* is a key component in Wnt-pathway as mentioned in the text. As an adherens junction protein, it is usually anchored inside the cellular member when inactive. Due to two proteins’ co-location in the area of cellular membrane, we speculated thatβ-catenin may mask *PLA2G4A*’s activity under some way to prevent its AA release at HR stage.

Gene pair *PLA2G5* and *PCAF* has differential co-expression pattern, the coefficient is -0.4797 at HR stage and 0.0076 at HS stage. The protein encoded by *PLA2G5* belongs to the secretory phospholipase A2 family, which release AA. The protein encoded by *PCAF* has histone acetyl transferase activity, in addition, it also has been suggested to possess an intrinsic ubiquitination activity (Linares *et al.*, 2007). Therefore, we suggest that in HR stage, *PLA2G5* may be the target of *PCAF*, and through intrinsic ubiquitination, *PCAF* may degrade itself to repress *PLA2G5*’s transcription.

Gene pair *PLA2G12A* and *CROT* has differential co-expression pattern. At HR stage, the coefficient is 0.5363, and at HS stage is 0. The protein encoded by *PLA2G12A* belongs to the secretory phospholipase A2 family. However, cellular function of *PLA2G12A* has not been fully investigated yet. One research (Murakami *et al.*, 2003) showed the protein encoded by *PLA2G12A* has a weak enzymatic activity *in vitro* and minimally affects cellular AA release. *CROT* encodes a carnitine acyltransferase that catalyzes the reversible transfer of fatty acyl groups between CoA and carnitine, it provides a crucial step in the transport of acyl-CoA out of peroxisome to cytosol and mitochondria. In virtue of the transfer character of *CROT*, we suggest that *CROT* may help the atypical member *PLA2G12A* of *PLA2* family to accomplish its function of AA release.

Gene pair *PTGS1* and *ALOX5* has differential co-expression pattern, and the coefficient is 0.6789 at HR stage and -0.0192 at HS stage. *PTGS1* encodes a protein which is usually termed as cyclooxygenase-1 (COX-1), and can catalyze a key step in converting the released AA to an unstable endoperoxide intermediate, PGH2, which can form prostanglandins (Nie *et al.*, 2001). *ALOX5* encodes protein which is usually entitled as lipoxygenase-5 (LOX-5), which can catalyze the released AA to produce hydroxyeicosatetraenoic acids (HETEs), which have bioactive effects for promoting prostate cancer (Nie *et al.*, 2001). Since *PTGS1* and *ALOX5* both play the key roles in released AA oxidation, the high positive correlation of them in HR stage may reflect that both the COX-mediated and LOX -mediated AA metabolism take effects at HR stage.

Gene pair *CEBPD* and *CTNNB1* shows high correlation at HR stage, yet shows little or no correlation at HS stage, with coefficient values altering from -0.0070 at HS stage to -0.6033 at HR stage. *CEBPD* encodes a protein which belongs to the basic-leucine zipper class of transcription factors, and plays an important role in adipogenesis to promote preadipocyte’s differentiation. The protein encoded by *CTNNB1*, usually entitled as *β*-catenin, plays a key role in the Wnt signalling, which has been reported to maintain preadipocytes in an undifferentiated state through inhibition *CEBP*s (Sarah *et al.*, 2000). Compared with the HS stage, the HR stage is considered poor differentiated or undifferentiated and *CEBPD* is subject to the inhibition of *CTNNB1*. Therefore, the pair shows much more negative correlation at the HR stage than at the HS stage.

Gene pair *PPARD* and *DVL1* is differentially co-expressed, and the coefficient is 0.5618 at HR stage and 0.0003 at HS stage. *PPARD* belongs to the *PPAR* family, and has been reported to show contradictory effects on cell destiny. Jarvis *et al.* (2005) has reported it as rescuing prostate epithelial cells from growth inhibition. *DVL1* encodes a protein which is a component in Wnt signaling and can inactivate *GSK3B*, prevent it from phosphorylating*β*-catenin with subsequently degradation. Because *PPARD* has been suggested to play a role through activation of downstream genes (Suchanek *et al.*, 2002), it may be assumed that *PPARD* can enhance *DVL1*’s transcription at HR stage with the positive correlation between *PPARD* and *DVL1*.

Gene pair *PPARD* and *NCOA2* has differential co-expression pattern, and the coefficient is -0.6462 at HR stage and -0.0191 at HS stage. The protein encoded by *NCOA2* possesses intrinsic histone acetyltransferase activity, can bind to the C-terminal transcriptional activation domain of nuclear hormone receptors, such as *PPARG*, and is required for the maximal *PPARG* activity (Rosen *et al.*, 2000). Adipogenesis is disadvantageous for a cell to maintain in undifferentiated state. Therefore, we suggest that at HR stage, *PPARD* may mask the activity of *NCOA2*, prevent it from enhancing activation of *PPARG*, which acts as an enhancing factor in adipogensis.

Based on the above interpretations of gene pairs, we infer the network of arachidonic acid metabolism during prostate cancer progression (illustrated in Figure 6).

Arachidonic acid is a major ingredient in animal fats and many vegetable oils, and after absorption it originally locates in cellular membrane glycerolipid pools. Usually, under the assistance of PLA2 family members, AA can be transported into cytoplasm and then its released form can be oxidized by COXs (shown as PTGS1 and PTGS2 in Figure 6) or LOXs (shown as ALOX5), to form a variety of bioactive eicosanoids (shown as PGJ2, PGE2, and HETEs; while PGH2 and PGD2 are unstable intermediates), which play great roles in tumor initiation, progression, and metastasis (see review Nie *et al.*, 2001).

PPARs are the downstream substrate of AA metabolism. As the biological interpretations described above, at HR stage, although PPARG is highly expressed, its activity is lost. This results from the inhibition of PPARD, which functions as a potent inhibitor of PPARG. The mechanism is shown in Figure 6. Products of released AA oxidation mediated by COXs, prostanglandins (shown as PGE2 and PGJ2 in Figure 6), are the ligands to activate PPAR family (Kliewee *et al.*, 1995; Wang *et al.*, 2004). To generate prostanglandins in promoting prostate cancer progression, both PTGS1 and PTGS2 are supposed to play important roles in HR stage. PTGS1 has been supposed to play role as well as ALOX5, according to our result mentioned before. PTGS2 has been reported by numerous studies in consensus that its expression is elevated during prostate cancer progression (Gupta *et al.*, 2000; Lee *et al.*, 2001; etc.), so it is also assumed to play role at HR stage.

In a holistic view, we speculate that there exists two conflicting powers — pro-deterioration and anti-deterioration—which are fighting and resisting each other during the prostate cancer progression from HS stage to HR stage. To prevent the deterioration trend, CTNNB1 and PCAF are recruited respectively to inhibit the activities of traditional PLA2 members PLA2G4A and PLA2G5, in order to repress eicosanoid producing. Oppositely, cancerous cells develop a scheme by recruiting CROT to help the atypical PLA2 member PLA2G12A to accomplish AA release. Moreover, for anti-deterioration, PPARG is initiated to arrest cancerous cell growth by inducing cell differentiation. On the other hand, cells employ PPARD to repress PPARG, which leads to the growth inhibition induced by PPARG being blocked.

**Tumor necrosis factor (TNF) signaling pathway.**

It has been reported that at the early stage of cancer, TNF plays the role as tumor repressor, but at the late stage, it can promote tumor cell proliferation (Balkwill, 2002). The mechanism for how the death-inducing capability of TNF to be occluded by concomitant activation of NF-κB still remains unclear. According to our results, the competitive relationship between TNF-induced NF-κB pathway and TNF-induced apoptosis pathway and the possible mechanism for TNF’s function alteration during prostate cancer progression could be partially revealed.

*Interpretations of gene-pairs in TNF-induced NF-κB pathway*

In our results, gene pair *PRKCA* and *IKBKB* has differential co-expression pattern, the coefficient is 0.7040 at HR stage and -0.0047 at HS stage. The protein encoded by *PRKCA* belongs to the protein kinase C (PKC) family, whose members can phosphorylate a wide variety of protein targets. The protein encoded by *IKBKB* is a catalytic subunit of I-κB kinase (IKK). IKK can phosphorylate I-κB to induce its ubiquitinated degradation, then NF-κB complex can be relieved from the mask of I-κB to get activated. IKK activation depends upon the phosphorylation of residues in the activation loop of IKBKB (Mukherjee *et al.*, 2006), and the events contributing to IKBKB phosphorylation have been not well understood. Therefore our results provide a conceivable mechanism for IKBKB phosphorylation caused by PRKCA.

Gene pair *PPP3R1* and *RELA* has differential co-expression pattern, the coefficient is -0.4975 at HS stage and -0.0032 at HR stage. The protein encoded by *PPP3R1* is a regulatory subunit of phosphatase 3, which has the dephosphorylation function. The protein encoded by *RELA* belongs to the Rel family (Verma *et al.*, 1995), whose members can form various dimers to constitute NF-κB as dimeric transcription factors. In mammal cell, the most common assembly for NF-κB is the heterodimer of p50 and RELA. Since the full obtaining for NF-κB activation depends on a further phosphorylation on RELA (Wang *et al.*, 2000), according to our finding, it seems that PPP3R1 may play the dephosphorylation role to inhibit NF-κB activation at HS stage.

Gene pair *ZA20D2* and *CSNK2A2* is differentially co-expressed, the coefficient is -0.5427 at HR stage and 0.0087 at HS stage. The protein encoded by *ZA20D2* plays an important role in the self-inhibitory circuit for NF-κB activation. Self-inhibitory circuit refers to the termination of signal-induced response. *ZA20D2* is triggered by overexpression of RIP and TRAF6 which are involved in TNF-induced NF-κB pathway, then it inhibits TNF-induced NF-κB activation in a dose-dependent manner, and does not inhibit TNF-induced apoptosis (Huang *et al.*, 2004). The protein encoded by *CSNK2A2* is a ubiquitous and pleiotropic Ser/Thr protein kinase involved in cell growth and transformation (Semplici *et al.*, 2002). Therefore, we suggest at HR stage, by phosphorylation and subsequential ubiquitination, CSNK2A2 could suppress the activity of ZA20D2, preventing it from attenuating TNF-induced NF-κB response.

*Interpretations of gene-pairs in TNF-induced apoptosis pathway*

In our results, gene pair *FAS* and *FADD* is differentially co-expressed with the coefficient changing from 0.0135 at HS stage to 0.4859 at HR stage. *FAS* encodes a death receptor which belongs to the TNF receptor superfamily that can induce apoptosis. *FADD* encodes death domain-containing adaptor, which can constitute a death-inducing signalling complex (DISC) with caspase-8 and -10 via the death effector domains (Muzio *et al.*, 1996). There are genetic evidences that all death receptors investigated for apoptosis so far critically depend on DISC (Sprick *et al.*, 2000; *etc.*), and activated receptor FAS can recruit FADD by homophilic interaction (Chinnaiyan *et al.*, 1995). The sample we used for androgen-dependent prostate cancer is untreated, i.e. the cancerous cell has been still fed with androgen; on the other side, for androgen-independent prostate cancer, it has suffered androgen ablation, i.e. the prostate cancer cell has been deprived of ‘nutrition’. Therefore, much more apoptosis may be initiated at HR stage, which results in *FAS* and *FADD*’s high correlation at HR stage and low correlation at HS stage.

Gene pair *TNFRSF10B* and *BNIP3L* is differentially co-expressed. The coefficient is 0.5732 at HR stage and 0.0127 at HS stage. *TNFRSF10B* encodes the protein usually termed as TNF-related apoptosis-inducing ligand receptor 2 (TRAILR2), which can directly recruit FADD by homophilic interaction (Chinnaiyan *et al.*, 1995). *BNIP3L* encodes a proapoptotic protein (Fei *et al.*, 2004). Thus, the positive correlation between the two genes suggests the TRAIL receptor-mediated apoptosis has been initiated by TNF at HR stage.

Gene pair *BCL2L2* and *BID* is differentially co-expressed, the coefficient is -0.3084 at HR stage and 0.0055 at HS stage. Proteins encoded by *BCL2L2* and *BID* both belong to the BCL-2 family, whose members can form hetero- or homo-dimers and act as anti- or pro-apoptotic regulators. Expression of *BCL2L2* in cells has contribution to reduced cell apoptosis under cytotoxic conditions (Zhu *et al.*, 2004). *BID* encodes a death agonist that is a mediator of mitochondrial damage induced by casps-8 to triggers cytochrome *c* release (Weng *et al.*, 2005), and it is reported that protein BCL2L2 has physical binding to protein BID (Denisov *et al.*, 2006). At HR stage, apoptosis is initiated but most cells still survive without programmed death. It means that the pro-apoptosis process has been repressed, while the anti-apoptosis process has been enhanced at HR stage. Then we infer that BCL2L2 is recruited to bind BID to mask BID’s pro-apoptotic activity and block the initiated apoptosis at HR stage. However, at HS stage, little apoptosis has been initiated so that the activities of the pro-apoptotic factor BID and anti-apoptotic factor BCL2L2 are weak. This may be the reason that the pair reveals different co-expression between HR stage and HS stage.

Gene pair *BCL2L2* and *MCL1* is differentially co-expressed, the coefficient altering from -0.0109 at HS stage to -0.5760 at HR stage. The protein encoded by *MCL1* belongs to the BCL-2 family, and is regulated transcriptionally and post-transcriptionally, to produce alternatively spliced isoforms. The full-length product (isoform 1) results in enhanced cell survival (Zhou *et al.*, 1997); while the central exon-skipped product (isoform 2) promotes apoptosis and is death-inducing (Colin *et al.*, 2000). In usual cases, the two isoforms both exist; and in the vast majority of pathological cases, including non-small cell lung cancer (Song *et al.*, 2005), and hepatocellular carcinoma (Fleischer *et al.*, 2006), *etc.*, *MCL1* is considerably over-expressed and plays the anti-apoptotic role as the full-length product. Interestingly, in our results, we found *MCL1* is significantly down-expressed at HR stage compared to HS stage (p < 0.01). Therefore, we suggest at HR stage, the prostate cancerous cells have been deprived of androgen, the splicing for *MCL1* is mainly performed in the isoform 2 way, i.e. central exon-skipped, to promote apoptosis. And we infer that at HR stage, cells prevent *MCL1* from promoting apoptosis in two manners. One is suppressing *MCL1*’s expression, which results in *MCL1* being significantly lowly expressed at HR stage. The other is recruiting BCL2L2 to form heterodimers with MCL1 to mask MCL1 pro-apoptotic activity.

Gene pair *BIRC2* and *MCL1* is differentially co-expressed, the coefficient is -0.4410 at HR stage and -0.0032 at HS stage. The protein encoded by *BIRC2* acts as E3 ubiquitin ligase (Yang *et al*., 2000). Since *MCL1* has been conceived to be shorted spliced to promote apoptosis, we suggest that BIRC2 may ubquitate MCL1, to promote its proteolytic process.

Gene pair *BIRC2* and *CDKN2A* is differentially co-expressed, the coefficient is -0.7492 at HR stage and -0.0005 at HS stage. Since *CDKN2A* is known to be an important tumor suppressor gene, has a functionality in cell cycle G1 control, our finding suggests during prostate cancer’s deterioration, the cyclin-dependent kinase inhibitor CDKN2A may be degraded by E3 ubiquitin ligase BIRC2, and the cell cycle for proliferation could go on.

In addition, gene pair *TNFAIP3* and *GSK3B* is differentially co-expressed. The coefficient is -0.6698 at HS stage and 0.0082 at HR stage. *TNFAIP3* is identified as a gene whose expression is rapidly induced by TNF, it encodes a zinc finger protein, and has been shown critical for inhibiting NF-κB activation (Lee *et al.*, 2000) as well as TNF-R1- mediated apoptosis (He and Ting, 2002). *GSK3B* encodes a proline-directed serine-threonine kinase which can degrade*β*-catenin through phosphorylation (Pennisi, 1998). Thus, we suggest the inhibitor TNFAIP3 at HS stage may be phosphortlated by GSK3B then degraded by proteosome.

*Network inference for TNF-induced pathway*

Based on our results, we infer a network as illustrated in Figure 4. To activate NF-κB, TNF induces the stimulation for IKBKB’s activity, then leads proteolytic degradation of I-κB, thus liberates NF-κB from I-κB’s mask, and allows NF-κB’s nuclear translocation to regulate the transcription of various downstream genes which are involved in suppressing apoptosis, and promoting chemoresistance as well as tumorigenesis (Bharti and Aggarwal, 2002). TNF-induced activation of IKBKB first requires TNF to stimulate TNFR1 (TNF receptor superfamily member 1), then the ligand-binded TNFR1 can recruit TRADD (death domain-containing adaptor), in order to serve together as an assembly platform for binding TRAF2 (TNF receptor- associated factor 2) and RIP (death domain-containing serine–threonine kinase). TRAF2 is sufficient to recruit IKBKB into TNFR1 complex whereas RIP is necessary for IKBKB activation (Devin *et al*., 2000). In the apoptosis pathway, TNFR1 can also act as a death receptor. Other death receptors such as Fas and TNFRSF10B directly bind to the death-inducing signaling complex (DISC) to initiate apoptosis, whereas TNFR1 is indirectly linked to FADD by TRADD, which is also responsible for bridging TNFR1 to both TRAF2 and IKBKB to activate NF-κB. We also suggest that both anti-deterioration and pro-deterioration mechanisms mainly function in this pathway. In HR stage, FAS- and TNFRSF10B- mediated apoptosis has been initiated to alleviate the deterioration; simultaneously, the already initiated apoptosis program may be blocked in various schemes including cell cycle turbulence, as illustrated in Figure 4.

In addition, we infer that both pathways of TNFR1-IKK and TNFR1-DISC are connected by self-inhibitory circuits, which may be responsible for the predominant in vivo specification of TNFR1 as an NF-κB-inducing receptor or as a death inducing receptor. Self-inhibitory circuit refers to the termination of TNF-induced response. ZA20D2 and TNFAIP3 are the key players in this mechanism. TNFAIP3 can inhibit both NF-κB activation and TNFR1-mediated apoptosis, whereas ZA20D2 only inhibits TNF-induced NF-κB activation (He and Ting, 2002). It is observed in our results that in HR stage, ZA20D2 reveals negative correlation with gene CSNK2A2. Then we infer that at HR stage, ZA20D2 may be degraded by CSNK2A2 as explicated earlier. Once TNF stimulates, because the inhibition of ZA20D2 is lost, NF-κB activation is predominant in the competition with FADD complex inducing apoptosis. Besides, TNFAIP3 shows highly negative correlation with GSK3B at HS stage but low co-expression at HR stage. We suggest that TNFAIP3 may be degraded by GSK3B. Apoptosis has not been initiated at HS stage in that the androgen-dependent tumor samples are untreated, i.e. the androgen has not been deprived. So at HS stage, in the competition for TNF participating in NF-κB activation and apoptosis, NF-κB prevails. However, finally, NF-κB will be inactivated by PPP3R1’s dephosphorylation on RELA to prevent the cancer aggravation.

**Androgen receptor pathway**

Androgens, acting via their receptors, regulate the development and maintenance of the differentiated function of the male reproductive system (Yan *et al.*, 1992; etc), and aberrant androgen receptor (AR) signaling is postulated to be an important mechanism for prostate cancer progression from androgen dependence to androgen independence (Taplin and Balk, 2004).

In our results, gene pair *PTGS1* and *HSD11B1* has differential co-expression pattern, the coefficient is 0.4027 at HR stage and 0.0015 at HS stage. The protein encoded by *HSD11B1* is a microsomal enzyme that can catalyze the conversion of the stress hormone cortisol to the inactive metabolite cortisone, or catalyze the reverse reaction. Since it has been reported that a double mutation in the androgen receptor allows prostate cancer cells to become responsive to cortisol (Brinkmann and Trapman, 2000), and usually *AR* gene at HR stage may probably get mutations (Navarre *et al*., 2002), we speculate that PTGS1 may activate HSD11B1, to make it produce active cortisol to promote prostate cancer cell growth.

Gene pair *BAG1* and *SVIL* are differentially co-expressed, the coefficient is -0.0017 at HS stage and 0.4024 at HR stage. The protein encoded by *BAG1* binds to BCL2 and enhances the anti-apoptotic effects of BCL2. Increased cytosolic and nuclear BAG1 expression may denote more aggressive prostate cancer (Krajewska *et al.*, 2006). Supervillin (SV) encoded by *SVIL* is a bipartite protein with distinct N- and C-terminal domains, the N-terminus contains nuclear localization signals and the C-terminus contains sequences similar to actin-binding proteins. Therefore, we infer that SV may bind to actins, and transport its ‘cargo’ into nucleus. Furthermore, SV has also been demonstrated to associate with AR and enhance its transcriptional activity in a ligand-induction manner (Ting *et al.*, 2002). So we suggest BAG1 may bind to SVIL and enhance its activity, so that the mutated AR could be transported into nucleus by SVIL with the cortisol induction.

Gene pair *HDAC1* and *PLAGL1* are differentially co-expressed, the coefficient is -0.8818 at HR stage and -0.0255 at HS stage. The protein encoded by *HDAC1* belongs to histone deacetylase family and can form complex with AR to repress its transcription activity (Gaughan *et al.*, 2002). *PLAGL1* encodes a zinc finger protein with transactivation and DNA-binding activity by increasing histone and acetyl coenzyme A affinities and catalytic activity (Hoffmann *et al.*, 2006). In addition, *PLAGL1* is expressed significantly higher at HR stage compared to HS stage with the p value 0.0105 for *T*-test. Accordingly, we infer that PLAGL1 may neutralize HDAC1’s repressive effect on AR, and promote AR’s transactivation at HR stage.

Gene pair *NCOA3* and *PA2G4* has differential co-expression pattern, the coefficient is -0.5250 at HR stage and -0.0087 at HS stage. The protein encoded by *NCOA3* has histone acetyltransferase activity, and is a coactivator for nuclear hormone receptor with binding to the receptor’s C-terminal transcriptional activation domain. The protein encoded by *PA2G4* acts as a repressor for the transactivation of androgen receptor by directly binding to androgen response elements within AR-regulated promoters and recruiting histone deacetylase HDACs to repress transcription (Zhang *et al*., 2005). Thus, our results suggest at HR stage, NCOA3 may block the repressing behaviors of PA2G2 on AR, which leads to the overexpression of prostate related genes.

Based on the above interpretations, we infer a network for AR-signaling as illustrated in Figure 7. We suppose that the aberrances in AR signaling can be manifested in two steps. The first step is the activation of AR including its transport into nucleus without androgen induction, and the second step is the regulation of nuclear coactivators and repressors for AR’s transactivation.

For genes *PTGS1* and *HSD11B1* showing positive co-expression only at HR stage, we suggest that *PTGS1* may promote *HSD11B1* to catalyze the conversion of the inactive metabolite cortisone to the stress hormone cortisol at HR stage. Then cortisol may activate AR without androgen induction, because it has been reported that a double mutation in the androgen receptor allows prostate cancer cells to become responsive to cortisol (Brinkmann and Trapman, 2000), and usually AR may probably get mutations at HR stage (Navarre *et al*., 2002). Then under the assistance of BAG1, SVIL may enhance AR’s activity and transport it into nucleus, as demonstrated in Figure 7.

In nucleus, the histone deacetylase family member HDAC1 can form complex with AR to repress its activity, and according to our result, it is suggested that at HR stage, this repression may get blocked because of PLAGL1’s neutralization effect on HDAC1. PA2G4 acts as a repressor for AR’s transactivation by directly binding to AR response elements within AR-regulated promoters and can recruit histone deacetylase HDACs to repress transcription. Histone acetyltransferase NCOA3, as a coactivator of AR, is required for proliferation of androgen-dependent and -independent cells (Zou *et al.*, 2006). Since it is shown in our result that NCOA3 and PA2G4 co-express negatively at HR stage, we suppose that the repressing effect of PA2G4 is blocked by NCOA3 at HR stage. After the two steps from cytoplasm to nucleus shown in Figure 7, the target genes of AR for prostate cell survival and masculine maintenance can be activated at HR stage.

**References**

Bharti, A.C. and Aggarwal,B.B. (2002) Nuclear factor-kappa B and cancer: its role in prevention and therapy. *Biochem. Pharmacol.*, **64**, 883–888.

Brinkmann,A.O. and Trapman,J. (2000) Prostate cancer schemes for androgen escape. *Nature Medicine*, **6**, 628–629.

Chinnaiyan,A.M. *et al.* (1995) FADD, a novel death domain-containing protein, interacts with the death domain of Fas and initiates apoptosis. *Cell*, **81**, 505–512.

Devin,A. *et al*. (2000) The distinct roles of TRAF2 and RIP in IKK activation by TNF-R1: TRAF2 recruits IKK to TNF-R1 while RIP mediates IKK activation. *Immunity*, **12**, 419–429.

[Fei,P](http://www.ncbi.nlm.nih.gov/sites/entrez?Db=PubMed&Cmd=Search&Term="Fei P"%5BAuthor%5D&itool=EntrezSystem2.PEntrez.Pubmed.Pubmed_ResultsPanel.Pubmed_RVAbstractPlus)**.** *et al.* (2004) Bnip3L is induced by p53 under hypoxia, and its knockdown promotes tumor growth. [*Cancer Cell*,](javascript:AL_get(this, 'jour', 'Cancer Cell.');) **6**, 597–609.

Gaughan,L. *et al.* (2002) Tip60 and histone deacetylase 1 regulate androgen receptor activity through changes to the acetylation status of the receptor. *J. Biol. Chem.*, **277**, 25904–25913.

Gupta,S. *et al.* (2000) Over-expression of cyclooxygenase-2 in human prostate adenocarcinoma. *Prostate*, **42**, 73–78.

He,K.L. and Ting,A.T. (2002) A20 inhibits tumor necrosis factor (TNF) alpha-induced apoptosis by disrupting recruitment of TRADD and RIP to the TNF receptor 1 complex in Jurkat T Cells. *Mol. Cell. Biol.*, **22,** 6034–6045.

Hoffmann,A. *et al.* (2006) Multitasking C2H2 zinc fingers link Zac DNA binding to coordinated regulation of p300-histone acetyltransferase activity. *Mol. Cell Biol.*, **26**, 5544–5557.

Huang,J. *et al.* (2004) ZNF216 is an A20-like and IκB kinase-interacting inhibitor of NF-κB activation. *J. Biol. Chem.*, **279**, 16847–16853.

Kliewee,S.A. *et al.* (1995) A Prostaglandin J_2_ Metabolite Binds Peroxisome Proliferator-Activated Receptor gamma and Promote Adipocyte Differentiation. *Cell*, **83**, 813–819.

Krajewska,M. *et al.* (2006) Expression of BAG-1 protein correlates with aggressive behavior of prostate cancers. *Prostate*, **66**, 801–810.

Lee,E.G. *et al.* (2000) Failure to regulate TNF-induced NF-κB and cell death responses in A20-Deficient mice. *Science,* **289,** 2350–2354.

Lee,L.M. *et al.* (2001) Expression of cyclooxygenase-2 in prostate adenocarcinoma and benign prostatic hyperplasia. *Anticancer Res.*, **21**, 1291–1294.

Linares,L.K. *et al.* (2007) Intrinsic ubiquitination activity of PCAF controls the stability of the oncoprotein Hdm2. *Nat. Cell Biol.*, **9**, 331–338.

[Mukherjee,S](http://www.ncbi.nlm.nih.gov/sites/entrez?Db=PubMed&Cmd=Search&Term="Mukherjee S"%5BAuthor%5D&itool=EntrezSystem2.PEntrez.Pubmed.Pubmed_ResultsPanel.Pubmed_RVAbstractPlus). *et al.* (2006) Yersinia YopJ acetylates and inhibits kinase activation by blocking phosphorylation. [*Science*,](javascript:AL_get(this, 'jour', 'Science.');) 312, 1211–1214.

Murakami,M. *et al.* (2003) Cellular arachidonate-releasing function of novel classes of secretory phospholipase A2s (Groups III and XII). *J. Biol. Chem.*, 278, 10657–10667.

Navarre,D. *et al.* (2002) Transition to androgen-independence in prostatec ancer. J. Steroid Biochem. *Mole. Biol*., 81(3), 191–201.

Nie,D. *et al.* (2001) Role of eicosanoids in prostate cancer progression. *Cancer Metastasis Rev.*, 20, 195–206.

Pennisi,E. (1998) [How a growth control path takes a wrong turn to cancer.](http://www.ncbi.nlm.nih.gov/entrez/query.fcgi?cmd=Retrieve&db=PubMed&list_uids=9750112&dopt=Citation) *Science*, 281, 1438–1441.

[Rao,M.A](http://www.ncbi.nlm.nih.gov/sites/entrez?Db=PubMed&Cmd=Search&Term="Rao MA"%5BAuthor%5D&itool=EntrezSystem2.PEntrez.Pubmed.Pubmed_ResultsPanel.Pubmed_RVAbstractPlus). *et al.* (2002) RanBPM, a nuclear protein that interacts with and regulates transcriptional activity of androgen receptor and glucocorticoid receptor. [*Biol. Chem.*](javascript:AL_get(this, 'jour', 'J Biol Chem.');), 277, 48020–48027.

[Semplici,F](http://www.ncbi.nlm.nih.gov/sites/entrez?Db=PubMed&Cmd=Search&Term="Semplici F"%5BAuthor%5D&itool=EntrezSystem2.PEntrez.Pubmed.Pubmed_ResultsPanel.Pubmed_RVAbstractPlus). *et al.* (2002) CK2-dependent phosphorylation of the E2 ubiquitin conjugating enzyme UBC3B induces its interaction with beta-TrCP and enhances beta-catenin degradation. [*Oncogene*,](javascript:AL_get(this, 'jour', 'Oncogene.');) 21, 3978–3987.

Ting,H.J. *et al.* (2002) Supervillin associates with androgen receptor and modulates its transcriptional activity. *Proc. Natl. Acad. Sci.* *USA*, 99, 661–666.

Verma,I.M. *et al.* (1995) Rel/NF-kappa B/I kappa B family: intimate tales of association and dissociation. *Genes Dev.*, 9, 2723–2735.

Wang,D. *et al.* (2000) Tumor necrosis factor alpha-induced phosphorylation of RelA/p65 on Ser529 is controlled by casein kinase II. *J. Biol. Chem.*, 275, 32592–32597.

Wang,D.Z. *et al.* (2004) Prostaglandin E2 promotes colorectal adenoma growth via transactivation of the nuclear peroxisome proliferator-activated receptor δ. *Cancer Cell*, 6, 285–295.

Yang,Y. *et al.* (2000) Ubiquitin protein ligase activity of IAPs and their degradation in proteasomes in response to apoptotic stimuli. *Science*, 288, 874–877.

[Zhang,Y](http://www.ncbi.nlm.nih.gov/sites/entrez?Db=PubMed&Cmd=Search&Term="Zhang Y"%5BAuthor%5D&itool=EntrezSystem2.PEntrez.Pubmed.Pubmed_ResultsPanel.Pubmed_RVAbstractPlus). and [Hamburger,A.W](http://www.ncbi.nlm.nih.gov/sites/entrez?Db=PubMed&Cmd=Search&Term="Hamburger AW"%5BAuthor%5D&itool=EntrezSystem2.PEntrez.Pubmed.Pubmed_ResultsPanel.Pubmed_RVAbstractPlus). (2005, a) Specificity and heregulin regulation of Ebp1 (ErbB3 binding protein 1) mediated repression of androgen receptor signalling. [*Br. J. Cancer*,](javascript:AL_get(this, 'jour', 'Br J Cancer.');) 92, 140–146.

[Zhang,Y](http://www.ncbi.nlm.nih.gov/sites/entrez?Db=PubMed&Cmd=Search&Term="Zhang Y"%5BAuthor%5D&itool=EntrezSystem2.PEntrez.Pubmed.Pubmed_ResultsPanel.Pubmed_RVAbstractPlus). *et al.* (2005, b) The ErbB3-binding protein Ebp1 suppresses androgen receptor-mediated gene transcription and tumorigenesis of prostate cancer cells. [*Proc. Natl. Acad. Sci. USA*,](javascript:AL_get(this, 'jour', 'Proc Natl Acad Sci U S A.');) 102, 9890–9895.

[Zou,J.X](http://www.ncbi.nlm.nih.gov/sites/entrez?Db=pubmed&Cmd=Search&Term="Zou JX"%5BAuthor%5D&itool=EntrezSystem2.PEntrez.Pubmed.Pubmed_ResultsPanel.Pubmed_RVAbstractPlus). *et al.* (2006) ACTR/AIB1/SRC-3 and androgen receptor control prostate cancer cell proliferation and tumor growth through direct control of cell cycle genes. [*Prostate*,](javascript:AL_get(this, 'jour', 'Prostate.');) 66, 1474–1486.
